# *Foeniculum vulgare* Mill. Mitigates Scopolamine-Induced Cognitive Deficits via Antioxidant and Neuroprotective Mechanisms in Zebrafish

**DOI:** 10.3390/molecules30132858

**Published:** 2025-07-04

**Authors:** Ion Brinza, Razvan Stefan Boiangiu, Elena Todirascu-Ciornea, Lucian Hritcu, Gabriela Dumitru

**Affiliations:** 1 Faculty of Sciences, Lucian Blaga University of Sibiu, 550024 Sibiu, Romania; ion.brinza@ulbsibiu.ro; 2 Department of Biology, Faculty of Biology, Alexandru Ioan Cuza University of Iasi, 700506 Iasi, Romania; razvan.boiangiu@uaic.ro (R.S.B.); ciornea@uaic.ro (E.T.-C.); gabriela.dumitru@uaic.ro (G.D.)

**Keywords:** *Foeniculum vulgare* essential oil, oxidative stress, acetylcholinesterase, in silico analysis, Alzheimer’s disease, neuroprotection

## Abstract

*Foeniculum vulgare* Mill. (Apiaceae) is an aromatic medicinal plant known for its anti-inflammatory, antispasmodic, antiseptic, carminative, diuretic, and analgesic properties. This study aimed to investigate the effects of *F. vulgare* essential oil (FVEO; 25, 150, and 300 μL/L) on the cognitive performance and brain oxidative stress in a scopolamine (SCOP; 100 μM)-induced zebrafish model of cognitive impairment. Additionally, the pharmacokinetic properties and bioactivity profiles of the main FVEO constituents were predicted to be used in silico tools, including SwissADME, pkCSM, PASS online, and ADMETlab 2.0. Behavioral assays, novel tank diving test (NTT), Y-maze, and novel object recognition (NOR) test, were used to evaluate anxiety-like behavior, spatial memory, and recognition memory, respectively. Biochemical assessments of acetylcholinesterase (AChE) activity and oxidative stress biomarkers were also conducted. The results demonstrated that FVEO significantly improved cognitive performance in SCOP-treated zebrafish, normalized AChE activity, and reduced oxidative stress in the brain. These findings suggest the therapeutic potential of FVEO in ameliorating memory impairment and oxidative damage associated with neurodegenerative disorders such as Alzheimer’s disease (AD).

## 1. Introduction

Alzheimer’s disease (AD) is a progressive neurodegenerative disease that is the most common form of dementia worldwide [1]. It is primarily characterized by the accumulation of amyloid-β plaques, hyperphosphorylated tau tangles, oxidative stress, and neuroinflammation. These pathological hallmarks often co-occur with cholinergic dysfunction, forming a complex network of interacting mechanisms that exacerbate disease progression [2,3]. AD is a complex and multifactorial neurodegenerative disorder, characterized by cholinergic deficits, β-amyloid peptide aggregation, accumulation of hyperphosphorylated tau proteins, mitochondrial dysfunction, oxidative stress, and chronic neuroinflammation. Disruption of redox homeostasis and microglial activation contribute to synaptic loss and neuronal degeneration, while transcriptional dysregulation of key regulatory factors such as nuclear factor erythroid 2-related factor 2 (Nrf2), cAMP response element-binding protein (CREB), and brain-derived neurotrophic factor (BDNF) impairs neuronal resilience and synaptic plasticity [4,5,6].

The cholinergic system, particularly acetylcholine (ACh), plays a central role in the pathophysiology of AD and remains a critical target for therapeutic intervention [7,8,9]. In AD, cholinergic atrophy and cognitive decline are closely linked, and dysregulation of this system contributes to further neurodegenerative changes, including abnormal tau phosphorylation and enhanced neuroinflammatory responses [7]. Current therapeutic strategies for AD primarily aim to restore cholinergic transmission and reduce oxidative stress. Clinically approved drugs such as cholinesterase inhibitors (AChEIs) and glutamate receptor antagonists have shown modest efficacy in improving cognitive symptoms [8]. AChEIs increase synaptic ACh levels and thereby enhance neuronal function. Several classes of AChEIs have been developed, including synthetic analogs, hybrid compounds, and naturally derived inhibitors [9]. Widely used agents such as donepezil, rivastigmine, and galantamine have demonstrated moderate success in improving cognition, behavior, and activities of daily living in patients with mild to moderate AD [10]. However, these drugs are often associated with adverse effects, including gastrointestinal disturbances (nausea, vomiting, diarrhea) and cardiovascular complications such as bradycardia [11]. Consequently, there is a pressing need to identify new therapeutic candidates with improved efficacy and safety profiles.

Scopolamine (SCOP), a muscarinic receptor antagonist, is frequently employed in experimental models to induce cognitive deficits resembling those observed in AD. SCOP administration leads to cholinergic dysfunction, amyloid-β accumulation, tau hyperphosphorylation, and increased oxidative stress, key features implicated in AD pathology [12,13,14,15]. Although SCOP-based models are limited in their ability to fully replicate the human disease, they remain valuable tools for screening cognitive enhancers and neuroprotective agents.

*Foeniculum vulgare* Mill. (Apiaceae), commonly known as fennel, has been traditionally used for its therapeutic properties, including anti-inflammatory, antioxidant, antispasmodic, diuretic, and analgesic effects. While direct evidence linking fennel to dementia is limited, its bioactive compounds suggest potential neuroprotective effects [16,17]. Notably, fennel has demonstrated efficacy in promoting nerve regeneration and functional recovery following peripheral nerve injury, indicating its potential role in neural health [18]. The plant contains a range of pharmacologically active constituents such as anethole, fenchone, limonene, estragole, and *p*-coumaric acid, which exhibit antioxidant, anti-inflammatory, carminative, diuretic, and other beneficial properties [19,20]. Additionally, fennel seeds are rich in flavonoids, linalool, and fatty acids (e.g., palmitic and oleic acids), which contribute to their anti-inflammatory activity [21]. Even the less-utilized parts of the plant, such as stems and leaves, contain phenolic acids and glycosylated flavonoids capable of inhibiting COX-2, further underscoring their therapeutic potential [17].

The present study aimed to investigate the potential neuroprotective effects of *F. vulgare* essential oil (FVEO) in a SCOP-induced zebrafish model of cognitive impairment. Specifically, we evaluated the effects of FVEO on anxiety-like behavior, cognitive performance, AChE activity, and oxidative stress markers. Furthermore, the pharmacokinetic and toxicological properties of major FVEO constituents were predicted using integrated in silico approaches.

## 2. Results and Discussions

### 2.1. Pharmacokinetic Profiles of Scopolamine, Galantamine, Trans-Anethole, Camphor, Trans-Sabinene Hydrate, (+)-Carvone, and β-Pinene

Free online platforms such as SwissADME [22], pKCSM [23] and ADMETlab 2.0 [24] were employed for the theoretical assessment of ADMET and pharmacokinetic properties of SCOP, galantamine (GAL), and the major bioactive constituents identified in FVEO from our previous study [25]: *trans*-anethole (58.1%), camphor (21.3%), *trans*-sabinene hydrate (3.1%), (+)-carvone (7.8%), and *β*-pinene (2.6%). Physicochemical parameters, including molecular weight, number of heavy atoms, number of aromatic heavy atoms, octanol-water partition coefficient (LogP), fraction of sp^3^-hybridized carbon atoms (Fraction Csp^3^), number of rotatable bonds, hydrogen bond acceptors and donors, molar refractivity, surface area, and topological polar surface area (TPSA) were analyzed (Table 1) to characterize these compounds comprehensively.

Molecular weight impacts absorption, distribution, and elimination; the number of heavy atoms and aromatic heavy atoms reflects molecular complexity and ring structure. LogP is indicative of lipophilicity and affects solubility and membrane permeability. Fraction Csp^3^ denotes the saturation level and three-dimensionality of the molecule. The number of rotatable bonds relates to molecular flexibility, while hydrogen bond donors and acceptors influence interactions with biological targets. Molar refractivity provides insight into molecular volume and polarizability; surface area reflects spatial occupancy, and TPSA correlates with the compound’s ability to cross biological membranes [23,24,26,27,28].

Analysis of these pharmacokinetic and ADMET properties allowed for a comprehensive assessment of the drug-likeness and therapeutic potential of the selected compounds, highlighting key factors that could influence their pharmacological behavior. The evaluated compounds included SCOP, GAL, and the major constituents of FVEO: *trans*-anethole (58.1%), camphor (21.3%), *trans*-sabinene hydrate (3.1%), (+)-carvone (7.8%), and *β*-pinene (2.6%), as identified in our previous study [25]. ADMET predictions obtained from SwissADME, ADMETlab 2.0, and pKCSM revealed promising absorption, distribution, metabolism, excretion, and toxicity profiles (Table 2).

All compounds demonstrated high human intestinal absorption (HIA), with values ranging from 72.626% for SCOP to 94.994% for GAL, and between 94.786% and 97.702% for the FVEO constituents, exceeding the recommended minimum threshold of 30%, and suggesting efficient intestinal absorption. Among the analyzed compounds, only SCOP was identified as a substrate for P-glycoprotein (P-gp), which could limit its intracellular retention by promoting efflux. Nevertheless, all compounds were predicted to be P-gp inhibitors, supporting their potential for enhanced bioavailability.

The volume of distribution at steady state (VDss) predictions indicated that all compounds, including SCOP, GAL, and the FVEO components, exceeded the cutoff value of log VDss > −0.15, suggesting moderate to high systemic distribution. The predicted unbound fraction in plasma (Fu), which affects drug availability and target binding, was lowest for *trans*-anethole (0.266) and *β*-pinene (0.35), followed by GAL (0.36), SCOP (0.414), camphor (0.459), *trans*-sabinene hydrate (0.469), and (+)-carvone (0.53).

Blood–brain barrier (BBB) permeability assessments showed that SCOP and GAL had log BB values below the threshold of 0.1, indicating limited central nervous system (CNS) penetration. In contrast, all FVEO-derived compounds had log BB values between 0.529 and 0.818, exceeding the threshold of 0.3, suggesting favorable BBB permeability. Similarly, CNS penetration predictions (log PS) revealed poor permeability for SCOP (–3.031), while the remaining compounds showed moderate-to-good CNS access, with values above the threshold of −2.

Cytochrome P450 (CYP) interactions were also evaluated. Only SCOP and GAL were predicted to be substrates for CYP3A4, a key metabolic enzyme. None of the analyzed compounds were found to inhibit CYP1A2, indicating a potentially favorable metabolic profile with a low risk of CYP-mediated drug–drug interactions. Regarding excretion, all compounds exhibited acceptable predicted clearance values, supporting their potential for efficient elimination.

Toxicological predictions indicated that SCOP (−0.319), GAL (−0.423), and *β*-pinene (0.371) had the highest predicted human toxicity, all below the toxicity threshold of log mg/kg/day < 0.447. Camphor showed moderate toxicity (0.473), while *trans*-sabinene hydrate (0.637), (+)-carvone (0.775), and *trans*-anethole (0.824) exhibited lower toxicity profiles. Acute oral toxicity in rats (LD_50_) ranged from 1.653 mol/kg for camphor (highest toxicity) to 2.728 mol/kg for GAL (lowest toxicity). For chronic oral toxicity (LOAEL), SCOP and GAL showed the lowest tolerated doses (0.736 and 0.966 mg/kg/day, respectively), whereas (+)-carvone and trans-anethole exhibited the highest tolerable doses (2.28 and 2.217 mg/kg/day, respectively). Among the compounds studied, only GAL was predicted to pose a risk for hepatotoxicity. Regarding skin sensitization, SCOP and GAL were predicted to be non-sensitizing, whereas all FVEO constituents, except *β*-pinene, showed potential to trigger allergic reactions in susceptible individuals.

Overall, the ADMET profiles of the tested compounds revealed favorable pharmacokinetic characteristics and manageable toxicity, supporting their potential for further development as pharmacological agents.

The intestinal environment provides several key features that influence drug.

It is essential to recognize that essential oils are inherently complex phytochemical mixtures, composed of numerous volatile and semi-volatile constituents that may interact in synergistic, additive, or even antagonistic ways. These interactions can substantially modulate the individual pharmacological activities of the components, making the overall bioactivity of the whole extract more than the sum of its parts. As such, while in silico predictive models, such as PASS or SwissADME, offer valuable preliminary insights into the drug-likeness, target pathways, and safety profiles of individual compounds, they fall short in capturing the multifactorial and dynamic nature of these interactions. Therefore, any conclusions derived from computational analysis should be interpreted with caution and not be extrapolated directly to the in vivo context without appropriate experimental validation. Nonetheless, in silico profiling serves a supportive role by helping to identify potentially bioactive constituents and by guiding hypothesis generation for further pharmacological investigation.

Moreover, although computational ADME-Tox simulations predicted favorable pharmacokinetic properties, including oral bioavailability, BBB permeability, and low toxicity for the major constituents of FVEO, this study did not include in vivo validation of these pharmacokinetic or toxicological aspects. As a result, key parameters such as systemic absorption, distribution to target tissues (especially the CNS), metabolic stability, routes of excretion, and potential for systemic toxicity or bioaccumulation remain unexplored.

### 2.2. The Similarities Between the Pharmacokinetic Properties of the Compounds

The SwissADME web server was employed to theoretically predict the drug-likeness of the compounds investigated in this study (Table 3). This analysis included evaluation against key drug-likeness filters: Lipinski’s rule of five [29], Weber’s rule [30], Ghose’s rule [31], and Egan’s Rule [32]. Compounds that satisfied these criteria were considered the most promising candidates. Additionally, the bioavailability score, a critical factor in drug development, was assessed. This score reflects the probability that a compound will be orally bioavailable in humans and has been refined over time through extensive research conducted by leading pharmaceutical companies worldwide [33]. Notably, small molecules that violate more than one of these rules are more likely to exhibit poor bioavailability and limited therapeutic potential [27].

The predictive results indicate that only SCOP and GAL fully comply with all five pharmacokinetic filters—Lipinski, Ghose, Veber, Egan, and Muegge—without any significant violations. This comprehensive compliance suggests that SCOP and GAL possess favorable drug-like properties and may serve as promising candidates for further drug development. In contrast, the major natural constituents of FVEO, *trans*-anethole, camphor, *trans*-sabinene hydrate, (+)-carvone, and *β*-pinene exhibited at least one violation of Ghose’s rule and two violations of Muegge’s rule. Notably, none of the FVEO-derived compounds violated Veber’s or Egan’s rule, and only β-pinene showed a violation of Lipinski’s rule.

Despite these minor deviations, all compounds achieved an Abbott bioavailability score of 0.55, placing them in a moderate probability class for oral bioavailability. This suggests that while the natural compounds from FVEO may present some limitations regarding their pharmacokinetic profiles, they still possess potential as orally active agents. Nonetheless, these in silico predictions represent a preliminary evaluation. Experimental validation through in vitro and in vivo studies is essential to confirm the bioavailability and therapeutic feasibility of these compounds for future drug development.

### 2.3. Prediction of Activity Spectra for Scopolamine, Galantamine, Trans-Anethole, Camphor, Trans-Sabinene Hydrate, (+)-Carvone, and β-Pinene

The investigated compounds, SCOP, GAL, *trans*-anethole, camphor, *trans*-sabinene hydrate, (+)-carvone, and *β*-pinene were subjected to detailed in silico evaluations using web-based platforms to assess their bioactivity profiles and medicinal chemistry properties. The predicted bioactivity scores and relevant pharmacological features are summarized in Table 4. The Prediction of Activity Spectra for Substances (PASS) platform provided *Pa* (probability of activity) and *Pi* (probability of inactivity) values for various biological effects. A *Pa* value > 0.5 generally indicates a higher likelihood of the compound exhibiting a particular biological activity.

SCOP showed the highest predicted affinity as an antagonist of nicotinic acetylcholine receptors (nAChRs), although the *Pa* value remained below the significance threshold (*Pa* > 0.5). For neuromuscular blocking activity on ACh, *trans*-anethole was the only compound exceeding the threshold (*Pa* = 0.594), followed by *trans*-sabinene hydrate (*Pa* = 0.483) and SCOP (*Pa* = 0.351). Notably, SCOP was the only compound predicted to act as a cholinergic antagonist (*Pa* = 0.713, *Pi* = 0.004), in line with its known pharmacodynamic profile. SCOP was also predicted to influence neurotrophic factor expression (*Pa* = 0.113), while camphor showed a slightly higher potential in this regard (*Pa* = 0.498, *Pi* = 0.041).

Several FVEO constituents, camphor, *trans*-sabinene hydrate, (+)-carvone, and *β*-pinene were predicted to possess antidementia activity, with *Pa* values exceeding the threshold of 0.5. GAL and *trans*-anethole also showed potential antidementia activity, albeit with slightly lower confidence (*Pa* = 0.458 and 0.499, respectively). Conversely, SCOP was unlikely to exhibit antidementia effects (*Pa* = 0.285). However, SCOP demonstrated a high probability of inducing delirium (*Pa* = 0.900) and behavioral disturbances (*Pa* = 0.861), consistent with its anticholinergic activity. It also showed moderate potential to inhibit lipid peroxidase (*Pa* = 0.521) and NADPH peroxidase (*Pa* = 0.431).

Among FVEO compounds, *β*-pinene (*Pa* = 0.521), camphor (*Pa* = 0.495), and *trans*-anethole (*Pa* = 0.485) were predicted to have antineurotic properties. Anti-inflammatory potential was observed for trans-sabinene hydrate (*Pa* = 0.839), *β*-pinene (*Pa* = 0.611), and *trans*-anethole (*Pa* = 0.526). Interestingly, GAL and *trans*-sabinene hydrate showed a moderate likelihood of inducing delirium (*Pa* = 0.612 and 0.622, respectively), and all tested compounds tended to cause behavioral disturbances. Additionally, GAL (*Pa* = 0.644), *trans*-anethole (*Pa* = 0.487), *trans*-sabinene hydrate (*Pa* = 0.450), and *β*-pinene (*Pa* = 0.462) displayed potential antidepressant activity. Of all compounds, only *trans*-anethole was predicted to inhibit both lipid peroxidase (*Pa* = 0.439) and NADPH peroxidase (*Pa* = 0.488), indicating potential antioxidant properties.

These results provide a preliminary silico framework supporting the pharmacological relevance of the tested compounds, particularly those derived from FVEO. However, experimental validation remains essential to confirm their predicted bioactivities.

### 2.4. Effects of FVEO on Anxiety-like Behavior in NTT

**Figure 1:**
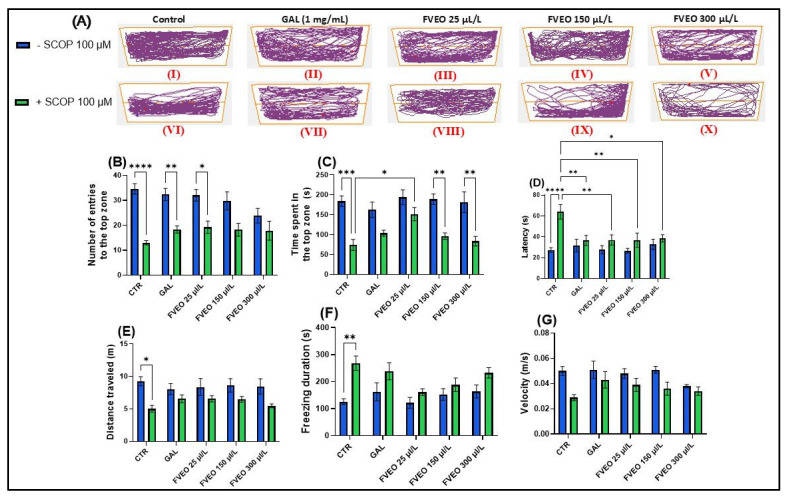
Effects of FVEO treatment (25, 150, and 300 μL/L) on scopolamine (SCOP, 100 µM)-exposed zebrafish in the novel tank test (NTT). Galantamine (GAL, 1 mg/L) was used as a positive control. (**A**) Schematic representation of zebrafish swimming patterns in the NTT; (**B**) number of entries into the top zone; (**C**) time spent in the top zone (s); (**D**) latency to first entry into the top zone (s); (**E**) distance traveled in the top zone (m); (**F**) freezing duration (s); (**G**) swimming velocity (m/s). Data are presented as mean ± S.E.M. (n = 10). Statistical analysis was performed using Tukey’s post hoc test: * *p* < 0.05, ** *p* < 0.001, *** *p* < 0.0001, and **** *p* < 0.00001.

To evaluate the effects of FVEO (25, 150, and 300 μL/L) on anxiety-like behavior in SCOP (100 μM)-exposed zebrafish using the novel tank test (NTT), specific behavioral parameters were recorded over a 6 min period per fish (Figure 1). These included the number of entries into the top zone, time spent in the top zone (s), and latency to enter the top zone (s) (Figure 1B–D). Additionally, to assess general locomotor activity and differentiate it from anxiety-specific responses, total distance traveled (m), freezing duration (s), and swimming velocity (m/s) were measured (Figure 1E–G).

In Figure 1A, the effects of treatment with SCOP (100 μM) and FVEO (25, 150, and 300 μL/L) on anxiety-like behavior in zebrafish are illustrated. The figure highlights locomotor behavior in both the top and bottom zones of the tank, providing insight into how these treatments influence vertical exploration and anxiety-related responses. Notably, zebrafish exposed to different FVEO concentrations displayed distinct behavioral patterns, suggesting dose-dependent modulation of anxiety-like behavior.

A one-way ANOVA revealed a significant main effect of treatment on the number of entries into the top zone (F(5, 54) = 2.33, *p* < 0.0001) (Figure 1B), with SCOP-treated zebrafish (group VI) showing a markedly reduced number of entries compared to the control group (group I). Similarly, there was a significant main effect of treatment on the time spent in the top zone (F(5, 54) = 1.61, *p* < 0.001) (Figure 1C), with SCOP-exposed fish spending less time in the top zone of the tank. Latency to first entry into the top zone was also significantly affected by the treatment (F(5, 54) = 3.70, *p* < 0.0001) (Figure 1D), with SCOP-treated zebrafish showing increased latency, further indicating heightened anxiety-like behavior.

In addition to vertical exploration, SCOP significantly altered locomotor activity. Total distance traveled was reduced (F(5, 54) = 0.93, *p* < 0.05) (Figure 1E), while freezing duration increased (F(5, 54) = 1.66, *p* < 0.01) (Figure 1F), and average velocity decreased (F(5, 54) = 1.10, *p* < 0.05) (Figure 1G). These results demonstrate that SCOP exerts a pronounced anxiogenic effect in zebrafish, reflected by impaired exploration and reduced locomotor activity.

Despite its anxiogenic effects in preclinical models, it is important to note that SCOP has demonstrated anxiolytic and antidepressant properties in clinical settings. In human populations diagnosed with depression and bipolar disorder, SCOP has shown efficacy in reducing depressive symptoms and has been associated with significant anxiolytic effects [33]. In contrast, findings from rodent models have been inconsistent. For example, Hughes and Otto [34], reported that SCOP-treated rats displayed increased anxiety levels in behavioral tests such as the open field and light/dark test assays. Interestingly, in zebrafish, Hamilton et al. [35] observed that treatment with a high concentration of SCOP (800 μM) increased exploration of the top zone in the NTT, suggesting anxiolytic-like effects. However, other studies have consistently identified SCOP as a robust model for anxiety and cognitive impairment in zebrafish, making it a reliable tool for neurobehavioral assessment [36,37,38,39,40]. These discrepancies highlight the complexity of SCOP’s neuropharmacological profile, which appears to be influenced by species differences, dosage, administration route, and exposure duration. In the present study, administration of FVEO to zebrafish acutely exposed to SCOP significantly modulated anxiety-like behavior. Notably, only the lowest FVEO concentration (25 μL/L) resulted in a significant increase in the number of entries into the top zone (*p* < 0.01), resembling the effect observed with GAL treatment (Figure 1B). Similarly, only zebrafish in group VIII (25 μL/L FVEO) showed a significant increase in time spent in the top zone (*p* < 0.05) (Figure 1C). In contrast, latency to first entry into the top zone significantly increased at all tested FVEO concentrations (*p* < 0.01 for 25 and 150 μL/L, *p* < 0.05 for 300 μL/L), compared to the SCOP-only group (VI) (Figure 1D). Importantly, chronic exposure to FVEO (25, 150, and 300 μL/L for 9 days) did not significantly affect total distance traveled (Figure 1E), freezing duration (Figure 1F), or average velocity (Figure 1G), indicating that FVEO does not impair general locomotor function. Our previous findings in a rodent model of AD [26] further support the anxiolytic potential of FVEO. In that study, inhalation of FVEO (1% and 3%) for 60 min daily over 21 days significantly increased the time spent in the open arms of the elevated plus maze in Aβ (1–42)-treated Wistar rats, particularly at the higher concentration (3%). The observed differences between models may reflect species–specific sensitivities to FVEO constituents or divergent pharmacodynamic responses. It is plausible that zebrafish, due to their aquatic environment and gill-based exposure pathways, are more sensitive to higher FVEO concentrations, which may attenuate or reverse the anxiolytic effects observed at lower doses.

The anxiolytic and antidepressant properties of *F. vulgare* have also been corroborated in clinical and preclinical studies. Alvarado-García et al. [41], reported significant reductions in anxiety and depression using Zung’s Self-Rating Scales following administration of essential oils from *F. vulgare* aerial parts and seeds. Similarly, Raman et al. [42] demonstrated that FVEO and its major component, *trans*-anethole, reduced anxiety-like behavior in rats subjected to social isolation stress in the elevated plus maze. Moreover, Bahari et al. [43] suggested that *trans*-anethole may act as an anti-stress agent by inhibiting key hypothalamic stress pathways, such as corticotropin-releasing hormone (CRH) and calcitonin gene-related peptide (CGRP).

### 2.5. Effects of FVEO on Zebrafish Spatial Memory Assessed in the Y-Maze Test

**Figure 2:**
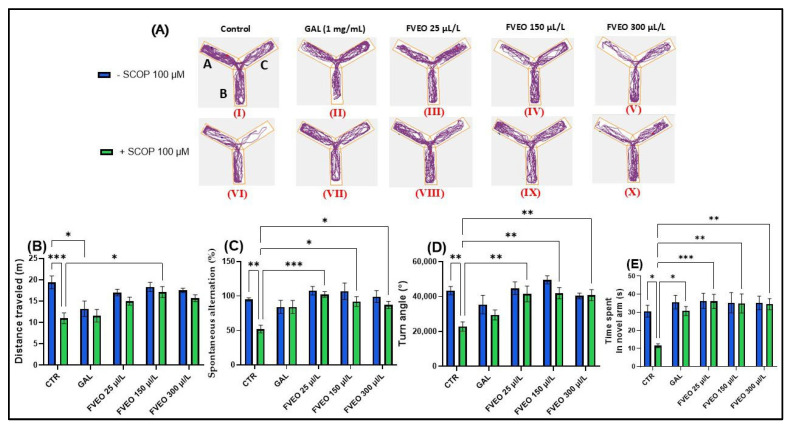
Effects of FVEO treatment (25, 150, and 300 µL/L) on scopolamine (SCOP, 100 µM)-induced cognitive impairment in zebrafish subjected to the Y-maze test. Galantamine (GAL, 1 mg/L) was used as a positive control. For Y-maze test: A—start arm; B—other arm and C—novel arm. (**A**) Representative locomotor trajectories during the second session of the Y-maze test; (**B**) distance traveled (m); (**C**) spontaneous alternation (%); (**D**) turn angle (°); (**E**) time spent in the novel arm (s). Data are presented as mean ± S.E.M. (n = 10). Statistical analysis was performed using Tukey’s post hoc test: * *p* < 0.05, ** *p* < 0.001, and *** *p* < 0.0001.

To investigate the effects of FVEO on spatial memory and novelty-induced behavior, zebrafish were subjected to the Y-maze test (Figure 2). The evaluated behavioral parameters included distance traveled (m), spontaneous alternation percentage (%), turn angle (°), and time spent in the novel arm (s). Figure 2A presents representative locomotor trajectories, highlighting the distinct movement patterns exhibited by zebrafish across the different experimental groups.

In the Y-maze, Tukey’s post hoc analyses revealed that SCOP-treated zebrafish (Group VI) showed a decrease in total distance traveled (*p* < 0.001) (Figure 2B) and a smaller turning angle (*p* < 0.01) (Figure 2D) compared to the control group (Group I), suggesting the induction of a hypolocomoric effect by SCOP. Simultaneously, group VI showed a lower percentage of spontaneous alternation (*p* < 0.01) (Figure 2C) and spent less time in the novel arm (*p* < 0.05) compared to the control group (Group I) (Figure 1E). These findings align with those reported by Volgin et al. [44], who showed that exposure of zebrafish to SCOP (100 µM) reduces the number of top zone entries in the NTT and the maximum velocity of zebrafish, thus suggesting hypoactive behavior and manifestations of anxiety. Similarly, Pande et al. [45], described hypolocomotor and amnesic effects of SCOP in zebrafish using the T-maze test. While the SCOP-induced zebrafish model provides a well-characterized platform for evaluating short-term cognitive impairment and neurochemical disruptions, it does not fully recapitulate the progressive and multifactorial nature of AD. In particular, the deficits induced by SCOP are acute and pharmacologically reversible, lacking the chronic neuropathological features such as amyloid-β deposition, tauopathy, and sustained neuroinflammation. Therefore, the findings reported here reflect the acute neuroprotective and antioxidant actions of FVEO and should be interpreted in the context of early-phase cognitive decline. Further validation in chronic mammalian models of AD is required to support translational applicability.

Although no significant behavioral changes were observed in zebrafish treated with FVEO alone (Groups II–V) compared to the control group (Group I), significant differences were found between the SCOP-only group (Group VI) and the FVEO + SCOP groups (Groups VII–X). FVEO treatment in SCOP-exposed zebrafish enhanced locomotor activity, as evidenced by increased total distance traveled (*p* < 0.05 for 150 µL/L; Figure 1B) and greater turn angle (*p* < 0.01 for 25, 150, and 300 µL/L; Figure 1D). Moreover, both GAL (1 mg/L) and FVEO (25, 150, and 300 µL/L) significantly improved exploratory behavior, as demonstrated by increased time spent in the novel arm (*p* < 0.05 for GAL; *p* < 0.001 for 25 µL/L FVEO; *p* < 0.01 for 150 and 300 µL/L FVEO; Figure 1E) and enhanced spontaneous alternation (*p* < 0.001 for 25 µL/L; *p* < 0.05 for 150 and 300 µL/L; Figure 1C), compared to SCOP-only zebrafish.

The memory-enhancing effects of *F. vulgare* have also been reported in previous studies. Delaram et al. [46] demonstrated that *F. vulgare* extract improved cognitive performance in an Aβ-induced rat model of AD. Similarly, Bhatti et al. [47] highlighted the neuroprotective potential of *F. vulgare* in a mouse model of lead-induced cerebral neurotoxicity, showing that treatment normalized the expression of amyloid precursor protein (APP) isoforms and oxidative stress markers.

### 2.6. Effects of FVEO on Recognition Memory Assessed in the NOR Test

**Figure 3:**
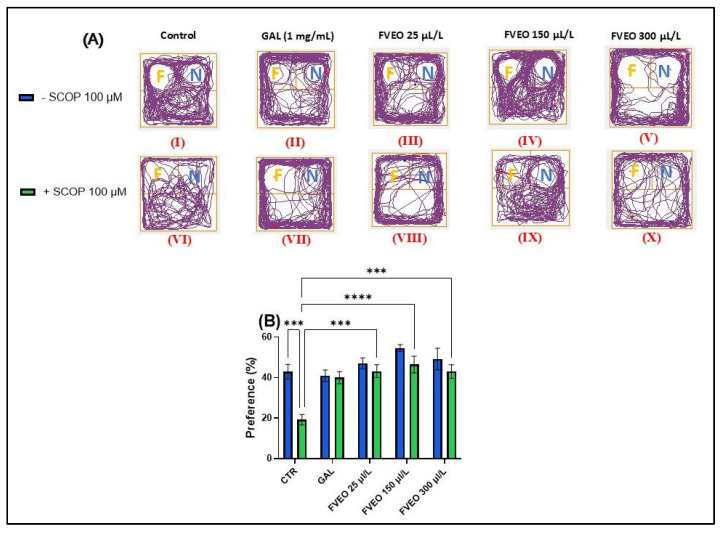
Effects of FVEO treatment (25, 150, and 300 µL/L) on recognition memory in scopolamine (SCOP, 100 µM)-treated zebrafish in the novel object recognition (NOR) test. Galantamine (GAL, 1 mg/L) was used as a positive control. (**A**) Representative locomotor trajectories during the NOR test session. The familiar object zone is indicated by “F” and the novel object zone by “N”; (**B**) percentage preference for the novel object. Data are presented as mean ± S.E.M. (n = 10). Statistical analysis was performed using Tukey’s post hoc test: *** *p* < 0.0001, and **** *p* < 0.00001.

To evaluate the effects of FVEO on recognition memory in acutely SCOP-treated zebrafish, the novel object recognition (NOR) test was employed. The test involved the use of complex geometric objects—specifically, a familiar yellow cube and a novel blue cube—to assess the ability of zebrafish to discriminate between familiar (F) and novel (N) stimuli. Figure 3A shows representative locomotor patterns recorded during the test across different experimental groups. Tukey’s post hoc analysis revealed a significant reduction in preference for the novel object in the SCOP-treated group (*p* < 0.001; Figure 3B) compared to the control group, indicating a SCOP-induced impairment in recognition memory. These findings are in line with our previous results demonstrating the amnesic effects of SCOP in zebrafish [48].

No statistically significant differences were observed between zebrafish treated with FVEO alone (Groups III–V) and the control group (Group I). However, a trend toward increased preference for the novel object was noted in the FVEO (150 µL/L)-treated group (Group IV), as shown in Figure 3B. In contrast, Tukey’s post hoc analysis revealed significant improvements in recognition memory in zebrafish chronically treated with FVEO (25, 150, and 300 µL/L) followed by acute SCOP administration (Groups VIII–X) compared to the SCOP-only group (Group VI). The most pronounced effects were observed at 25 and 150 µL/L FVEO (*p* < 0.0001), while 300 µL/L also produced a significant improvement (*p* < 0.001), as indicated by the increased preference for the novel object (Figure 3B).

These findings are consistent with previous reports. Memory-enhancing effects of *F. vulgare* extract (50, 100, and 200 mg/kg, administered orally) were demonstrated in a SCOP-induced Wistar rat model of cognitive impairment (1 mg/kg, i.p.) [49]. Additionally, Chang et al. [50] reported that *trans*-anethole, a major component of FVEO, facilitates long-term potentiation (LTP) through both NMDA receptor-dependent and -independent mechanisms, and mitigates trimethyltin-induced LTP impairment. These findings suggest that *trans*-anethole may regulate hippocampal synaptic plasticity and could have therapeutic potential for cognitive dysfunction associated with neurodegenerative diseases such as AD.

### 2.7. Effects of FVEO on AChE Activity

Cholinergic neurotransmission plays a central role in cognitive function, and its disruption is a hallmark of AD. In the early stages of AD, which typically present with progressive short-term memory loss, there is a selective degeneration of cholinergic neurons in the basal forebrain. Consequently, many pharmacological strategies for AD treatment focus on enhancing cholinergic function [51]. AChEIs are designed to mitigate the cholinergic deficit underlying cognitive and neuropsychiatric symptoms in AD by inhibiting the breakdown of ACh. ACh-positive neurons project widely to the cortex, modulating cortical processing and responses to novel stimuli. Beyond its role in terminating cholinergic neurotransmission, AChE has been implicated in broader pathophysiological processes, including the regulation of other proteins, regional cerebral blood flow, tau phosphorylation, and the amyloid cascade-factors that may influence AD progression [52].

**Figure 4:**
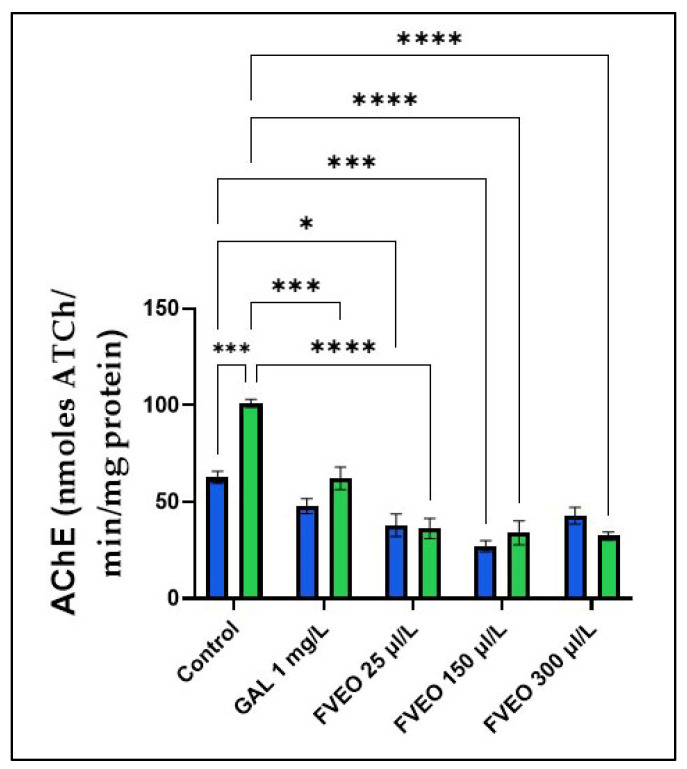
Effects of FVEO treatment (25, 150, and 300 µL/L) on acetylcholinesterase (AChE) specific activity in scopolamine (SCOP, 100 µM)-treated zebrafish. Galantamine (GAL, 1 mg/L) was used as a positive control. Data are expressed as mean ± S.E.M. (n = 10). Statistical significance was determined using Tukey’s post hoc test: * *p* < 0.05, *** *p* < 0.0001, and **** *p* < 0.00001.

To explore the potential mechanism by which FVEO (25, 150, and 300 µL/L) mitigates SCOP-induced memory deficits, AChE activity was assessed in zebrafish brain homogenates. As shown in Figure 4, SCOP-treated zebrafish (Group VI) exhibited a significant increase in AChE activity compared to the control group (Group I) (*p* < 0.001), indicating enhanced cholinergic breakdown and supporting the involvement of the cholinergic system in SCOP-induced cognitive impairment.

However, chronic administration of FVEO significantly reduced AChE activity in SCOP-treated zebrafish at all tested concentrations (25, 150, and 300 µL/L; *p* < 0.0001), compared to the SCOP-only group (Group VI). Notably, FVEO at 25 µL/L also modulated AChE activity when administered alone, suggesting a direct inhibitory effect. These findings indicate that FVEO may exert protective effects against SCOP-induced cholinergic dysfunction by modulating AChE activity.

Previous studies support these observations. Abdel-Baki et al. [53] reported the inhibitory effects of FVEO and its major components, *trans*-anethole and fenchone, on AChE activity in different developmental stages of *Musca domestica*. Similarly, Joshi et al. [54] demonstrated that an eight-day oral administration of methanolic extract from the whole plant of *F. vulgare* Linn. attenuated SCOP (0.4 mg/kg)-induced memory impairment and age-related cognitive decline in mice, partly through regulation of AChE activity. Additionally, *trans*-anethole has been shown to possess anti-AChE properties in *Ephestia kuehniella* Zeller [55], further supporting its potential role in modulating cholinergic pathways.

### 2.8. Effects of FVEO on the Antioxidant System Activity

Oxidative stress results from an imbalance between oxidants and antioxidants, favoring the accumulation of reactive species. This imbalance may stem from excessive free radical production or diminished antioxidant capacity. Molecular oxygen reduction sequentially generates superoxide anions and hydrogen peroxide, which can further convert into highly reactive hydroxyl radicals, collectively termed reactive oxygen species (ROS). These ROS interact with cellular macromolecules, including lipids, proteins, and nucleic acids, altering their structure and function [56]. The brain is particularly vulnerable to oxidative damage due to its high oxygen consumption, abundant lipid content, and limited antioxidant capacity. Oxidative damage in the brain is exacerbated by mitochondrial dysfunction, elevated metal ion concentrations, inflammation, and Aβ accumulation [57]. In AD, oxidative stress has been implicated as a key pathological event, affecting both characteristic lesions (senile plaques and neurofibrillary tangles) and morphologically normal neurons [58]. Consequently, oxidative stress is considered an early and central mechanism in AD pathogenesis, and antioxidant-based interventions may offer therapeutic potential [57].

**Figure 5:**
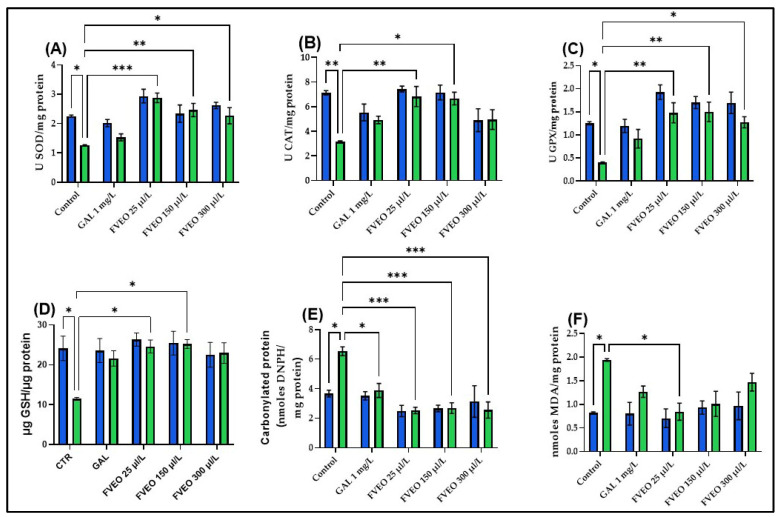
Effects of FVEO treatment (25, 150, and 300 µL/L) on oxidative stress markers in scopolamine (SCOP, 100 µM)-treated zebrafish. (**A**) Superoxide dismutase (SOD) activity; (**B**) catalase (CAT) activity; (**C**) glutathione peroxidase (GPX) activity; (**D**) reduced glutathione (GSH) levels; (**E**) protein carbonyl content; (**F**) malondialdehyde (MDA) levels. Galantamine (GAL, 1 mg/L) was used as a positive control. Data are expressed as mean ± S.E.M. (n = 10). Statistical significance was determined using Tukey’s post hoc test: * *p* < 0.05, ** *p* < 0.001, and *** *p* < 0.0001.

To explore the potential antioxidant effects of FVEO, the activities of key enzymatic antioxidants, superoxide dismutase (SOD), catalase (CAT), and glutathione peroxidase (GPX), were measured, along with reduced glutathione (GSH) content, protein carbonyl levels, and malondialdehyde (MDA) concentrations in zebrafish brain tissue. Acute SCOP exposure (100 µM, 30 min) significantly impaired oxidative status compared to control zebrafish (Group I), with reductions in SOD (*p* < 0.05; Figure 5A), CAT (*p* < 0.01; Figure 5B), and GPX activity (*p* < 0.05; Figure 5C), along with decreased GSH content (*p* < 0.05; Figure 5D). SCOP also increased oxidative damage, reflected by elevated carbonyl protein (*p* < 0.05; Figure 5E) and MDA levels (*p* < 0.05; Figure 5F). These results are consistent with previous studies showing that SCOP induces oxidative stress and disrupts the cholinergic system in both rodents [59,60] and zebrafish [40]. Additionally, SCOP has been shown to suppress Nrf2 expression and impair CREB/BDNF signaling, contributing to decreased synaptic plasticity and antioxidant defense [61,62,63].

No significant differences were observed between zebrafish treated with FVEO alone (Groups III–V) and the control group (Group I) regarding antioxidant enzyme activity or oxidative stress markers. However, FVEO treatment (25, 150, and 300 µL/L) significantly improved oxidative parameters in SCOP-treated zebrafish (Groups VIII–X). Specifically, SOD activity was restored at all concentrations (*p* < 0.001 for 25 µL/L, *p* < 0.01 for 150 µL/L, and *p* < 0.05 for 300 µL/L; Figure 5A). CAT activity was also significantly increased at 25 µL/L (*p* < 0.01) and 150 µL/L (*p* < 0.05), but not at 300 µL/L (Figure 5B). GPX activity was enhanced at all concentrations (*p* < 0.01 for 25 and 150 µL/L; *p* < 0.05 for 300 µL/L; Figure 5C). Additionally, GSH content was restored at 25 and 150 µL/L (*p* < 0.05; Figure 5D). FVEO significantly reduced carbonyl protein levels at all concentrations (*p* < 0.001), like the effect observed with GAL (*p* < 0.05; Figure 5E). Interestingly, only the lowest concentration of FVEO (25 µL/L) significantly attenuated MDA levels (*p* < 0.05), suggesting that lower doses may be more effective in preventing lipid peroxidation (Figure 5F).

The antioxidant and neuroprotective effects of FVEO observed in this study, reflected by the reduction in MDA levels and the restoration of endogenous antioxidant enzyme activities (SOD, CAT, GPx), suggest the involvement of cellular redox-regulating mechanisms. However, specific molecular pathways that may mediate these effects, such as the Nrf2–ARE signaling axis, known for its pivotal role in the transcriptional activation of antioxidant genes or the CREB-dependent pathway, implicated in neuroprotection, synaptic plasticity, and memory consolidation, were not explored [64].

It is important to note that, although the present data do not include direct molecular analyses, the existing literature provides supporting evidence for the involvement of the Nrf2-ARE axis in the effects of *F. vulgare*. For instance, in a UVB-induced skin photoaging model, extracts from *F. vulgare* have been shown to stimulate nuclear translocation of Nrf2 and enhance the expression of antioxidant enzymes such as GSH in human fibroblast cells and skin tissues of animal models, highlighting the activation of this cytoprotective pathway [65]. Therefore, it can be hypothesized that FVEO exerts its antioxidant effects in part through the activation of the Nrf2 signaling pathway. However, to confirm this mechanism in a neurodegenerative context, further studies incorporating gene expression profiling and proteomic analyses are warranted. Nonetheless, external evidence supports the modulation of this antioxidant axis by major FVEO constituents. For example, trans-anethole significantly activated Nrf2 and upregulated the expression of cytoprotective antioxidant enzymes such as SOD and GPx in the intestines of infected broiler chickens (600 mg/kg diet; *p* < 0.05), demonstrating a positive regulatory effect on the Nrf2-ARE pathway [66]. Similarly, α-pinene has been shown to modulate memory by activating the BDNF/TrkB/CREB signaling pathway in epilepsy models, indicating the potential of monoterpene compounds to influence neurotrophic signaling mechanisms [67]. Therefore, there is substantial evidence suggesting that despite the lack of direct experimental validation in the zebrafish model, the major constituents of FVEO may activate the Nrf2-ARE axis and, at least indirectly, modulate the CREB–BDNF pathway. The studies cited provide mechanistic support and a theoretical framework for interpreting our findings, particularly, the optimal effects observed at the lowest tested dose (25 µL/L) which may be attributed to hormesis and efficient activation of these stress-responsive intracellular signaling cascades.

These findings are supported by earlier studies. FVEO pretreatment has been shown to inhibit cyclophosphamide-induced cytotoxicity in mouse bone marrow cells by reducing micronuclei formation and enhancing antioxidant enzyme activities, including SOD, CAT, and GSH, while lowering MDA levels in the liver [68]. Barakat et al. [69] also reported that *F. vulgare* seeds and sprouts contain phenolic and volatile compounds capable of preventing oxidative DNA damage and ameliorating CCl_4_-induced hepatotoxicity in rats. Moreover, Imran et al. [70], demonstrated that *F. vulgare* extract promotes functional recovery and reduces oxidative stress in a mouse model of sciatic nerve injury.

### 2.9. Correlation Analyses Between Behavioral and Biochemical Parameters

**Figure 6:**
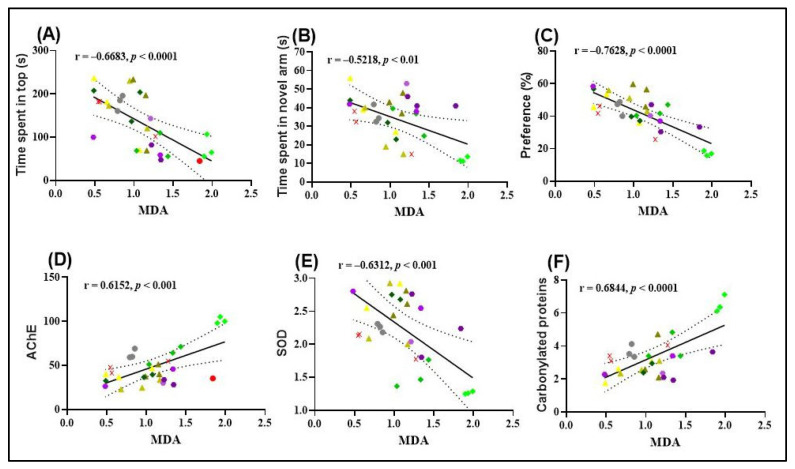
Pearson correlation analyses of behavioral and biochemical parameters in zebrafish. (**A**) Time spent in the top zone (NTT) vs. MDA levels (n = 10, r = –0.6683, *p* < 0.0001); (**B**) time spent in the novel arm (Y-maze) vs. MDA levels (n = 10, r = –0.5218, *p* < 0.01); (**C**) preference for the novel object (NOR test) vs. MDA levels (n = 10, r = –0.7628, *p* < 0.0001); (**D**) AChE activity vs. MDA levels (n = 10, r = 0.6152, *p* < 0.001); (**E**) SOD activity vs. MDA levels (n = 10, r = –0.6312, *p* < 0.001); (**F**) carbonylated protein levels vs. MDA levels (n = 10, r = 0.6844, *p* < 0.0001). Experimental groups include: (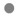) Control, (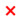) Galantamine (GAL, 1 mg/L), (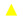) FVEO 25 µL/L, (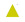) FVEO 150 µL/L, (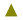) FVEO 300 µL/L, (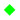) Scopolamine (SCOP, 100 µM), (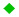) SCOP + GAL, (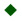) SCOP + FVEO 25 µL/L, (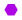) SCOP + FVEO 150 µL/L, and (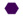) SCOP + FVEO 300 µL/L.

The results obtained from Pearson’s correlation analysis provide valuable insights into the relationships between behavioral outcomes and oxidative stress markers in zebrafish. A significant negative correlation was observed between the time spent in the top zone during the NTT and MDA levels (r = −0.6683, *p* < 0.0001; Figure 6A), suggesting that increased exploratory behavior is associated with reduced lipid peroxidation. This finding supports the hypothesis that FVEO may exert anxiolytic effects partly through its antioxidant properties.

Similarly, a significant negative correlation was found between the time spent in the novel arm of the Y-maze and MDA levels (r = −0.5218, *p* < 0.01; Figure 6B), indicating that enhanced spatial exploration correlates with lower oxidative damage. This further supports the role of FVEO in mitigating SCOP-induced oxidative stress and improving cognitive function.

A particularly strong negative correlation was observed between the percentage of preference for the novel object in the NOR test and MDA levels (r = −0.7620, *p* < 0.0001; Figure 6C), suggesting that improved recognition memory is associated with reduced lipid peroxidation. Collectively, these findings highlight a potential link between the behavioral improvements induced by FVEO and its antioxidative capacity, specifically in reducing MDA, a key marker of oxidative damage.

A significant negative correlation was also identified between SOD activity and MDA levels (r = −0.6683, *p* < 0.001; Figure 6E), suggesting that higher SOD activity is associated with reduced lipid peroxidation. This finding supports the potential protective role of SOD against oxidative damage. In contrast, AChE activity and the level of carbonylated proteins showed significant positive correlations with MDA levels. Specifically, a strong positive correlation was observed between AChE activity and MDA (r = 0.6152, *p* < 0.001; Figure 6D), as well as between carbonylated protein levels and MDA (r = 0.6844, *p* < 0.0001; Figure 6F). These results imply a possible link between increased oxidative stress, enhanced AChE activity, and protein oxidation, further supporting the hypothesis that lipid peroxidation is associated with cholinergic dysfunction and oxidative damage in SCOP-treated zebrafish.

These findings are in line with the previous literature demonstrating the neuroprotective and antioxidant effects of major FVEO constituents. Ryu et al. [71] reported that sabinene mitigates ROS-induced skeletal muscle atrophy via modulation of the MAPK/MuRF-1 pathway, indicating its capacity to counter oxidative stress. Dhingra et al. [72] demonstrated that *trans*-anethole possesses significant antidepressant activity in both stressed and non-stressed mice, likely mediated by inhibition of monoamine oxidase A (MAO-A), reduction in oxidative stress markers such as plasma nitrite and brain MDA, and increases in GSH and CAT levels. The reversal of corticosterone elevation also contributed to its antidepressant effects.

Similarly, Salama et al. [73] showed that camphor exerts antioxidant and anti-inflammatory effects by enhancing CAT and Nrf2 activity while reducing nitric oxide (NO), MDA, TNF-α, and TLR4 levels. Camphor also increased neurotransmitter levels (serotonin, dopamine, GABA) and neuronal signaling proteins in a model of ciprofloxacin-induced neurotoxicity. Dai et al. [74] reported that *d*-carvone significantly improved outcomes in a rat model of cerebral ischemia/reperfusion injury by reducing brain edema and infarct size, improving neurological scores, and exerting antioxidant, anti-inflammatory, and anti-apoptotic effects through modulation of pro-inflammatory cytokines and apoptotic pathways. Additionally, Dahiya et al. [75] demonstrated that *β*-pinene reversed cognitive impairment induced by intracerebroventricular streptozotocin (ICV-STZ) in mice, without affecting locomotor function. β-Pinene normalized mitochondrial enzyme activities (complexes I–IV), reduced AChE activity, and restored antioxidant parameters (GSH, CAT, SOD), while decreasing lipid peroxidation in the cortex and hippocampus.

Taken together, these findings suggest that FVEO may exert therapeutic effects through multifaceted mechanisms involving behavioral improvements and attenuation of oxidative stress. The observed correlations between behavioral performance, enzymatic antioxidant activity, and lipid peroxidation markers underscore the complexity of the neuroprotective effects of FVEO. Its impact on cholinergic function and oxidative balance positions it as a promising candidate for mitigating symptoms associated with amnesia and anxiety.

## 3. Materials and Methods

### 3.1. Estimated In Silico Pharmacokinetic Profile of Compounds

**Figure 7:**
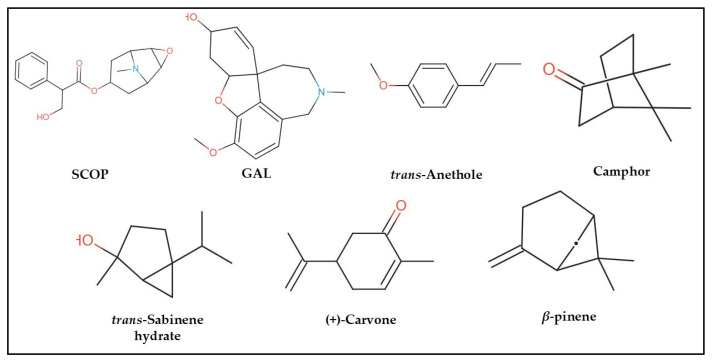
Chemical structures of scopolamine (SCOP), galantamine (GAL), and the main constituents of *Foeniculum vulgare* essential oil (FVEO): *β*-pinene, *trans*-sabinene hydrate, camphor, (+)-carvone, and *trans*-anethole.

In the computational analysis, we employed a simplified approach to represent chemical structures using the canonical Simplified Molecular Input Line Entry System (SMILES) notation for SCOP, GAL, and the principal constituents of FVEO, including *β*-pinene, *trans*-sabinene hydrate, camphor, (+)-carvone, and *trans*-anethole (Figure 7). The chemical composition of FVEO was previously characterized using an Agilent 6890 GC-MS system (Santa Clara, CA, USA) equipped with a split/splitless injector, as described in detail elsewhere [25]. Canonical SMILES data were retrieved from the PubChem database in November 2023.

To predict and evaluate the pharmacokinetic and physicochemical properties of these compounds, we used three freely available computational platforms: SwissADME [22], pKCSM [23], and ADMETlab 2.0 [24]. These tools provide in silico predictions for key pharmacological parameters such as solubility (log S), absorption, distribution, metabolism, excretion, and toxicity (ADMET). To ensure consistency across different platforms and to facilitate meaningful comparisons with experimental data, we standardized measurement units for parameters such as solubility and clearance. Additionally, parameters such as absorption and permeability were converted into binary categories (e.g., “Yes/No”) due to differing classification criteria used by the platforms. This standardized and methodical approach allowed for objective interpretation of predictive data, enhancing the reliability of comparisons between computational results and experimental findings, and supporting the evaluation of the pharmacological potential of the selected compounds.

The selected predictive parameters included: (1) human intestinal absorption, (2) P-glycoprotein substrate status, (3) P-glycoprotein I/II inhibition, (4) volume of distribution at steady state (VDss, human), (5) blood–brain barrier (BBB) permeability, (6) central nervous system (CNS) permeability, (7) CYP3A4 substrate status, (8) CYP1A2 inhibition, (9) total clearance, (10) maximum tolerated dose in humans, (11) oral acute toxicity in rats (LD_50_), (12) oral chronic toxicity in rats (LOAEL), and (13) hepatotoxicity and skin sensitization.

### 3.2. Prediction of the Biological Activity of the Compounds via PASS Online Platforms

The Prediction of Activity Spectra for Substances (PASS) online tool [76] was used to estimate the potential biological activities and adverse effects of the tested compounds. PASS predictions are based on structure–activity relationships, analyzing the correlation between a compound’s chemical structure and its known biological activities. The following activity probabilities were selected for evaluation: ACh nicotinic antagonist, ACh neuromuscular blocking agent, cholinergic antagonist, neurotransmitter antagonist, neurotrophic factor enhancer, dementia treatment, antineurotic activity, anti-inflammatory activity, anti-delirium effect, behavioral disturbance modulation, antidepressant activity, lipid peroxidase inhibition, and inhibition of nicotinamide adenine dinucleotide phosphate (NADPH) peroxidase.

### 3.3. Compound Prediction, Drug Analogy

To rigorously assess the drug-likeness and structural suitability of the investigated compounds, we employed the SwissADME online platform [22], which enables comprehensive in silico evaluation based on established medicinal chemistry filters. The following drug-likeness rules were applied: Weber’s rule [30], Lipinski’s rule of five [29], Egan’s rule [32], and Ghose’s rule [31]. Lipinski’s rule of five evaluates oral bioavailability by setting limits on key molecular properties: molecular weight (<500 Da), lipophilicity (Log P < 5), hydrogen bond donors (≤5), and hydrogen bond acceptors (≤10). Weber’s rule focuses on molecular flexibility and polar surface area, recommending a maximum of 10 rotatable bonds and a topological polar surface area (TPSA) of ≤ 140 Å. Ghose’s filter incorporates broader criteria, including molecular weight (160–480 Da), Log P (−0.4 to 5.6), molar refractivity (40–130), and total number of atoms (20–70), to define drug-likeness based on a wider physicochemical profile. Egan’s rule evaluates the potential for oral bioavailability based on Log P and TPSA values and provides insights into membrane permeability and absorption, also relevant for liver exposure and toxicity considerations.

Together, these structural filters help determine whether the compounds possess favorable physicochemical characteristics for further development as drug candidates and assess their safety and pharmacokinetic suitability in a medicinal context [32].

### 3.4. Experimental Design and Ethical Considerations

This study aimed to evaluate the neuropharmacological effects of FVEO on cognitive performance, anxiety-like behavior, and oxidative status in zebrafish (*Danio rerio*). A total of 100 adult short-fin Tubingen wild-type zebrafish (5–7 months old) with a 1:1 male-to-female ratio were used. The animals were obtained from the European Zebrafish Resource Center (Institute of Toxicology and Genetics, Germany) and acclimated under quarantine for two weeks. Following quarantine, zebrafish were housed in groups of 10 per a 10 L tank containing dechlorinated water treated with Tetra AquaSafe (Melle, Germany) and replaced daily.

Environmental parameters were rigorously controlled: water temperature was maintained at 28 ± 2 °C, pH at 7.0–8.0, dissolved oxygen at 8 ± 1 mg/L, conductivity at 1400–1500 µS/cm, and ammonia and nitrite concentrations were maintained below 0.001 mg/L. A 14:10 h light–dark cycle was used, and fish were fed Norwin Norvital flakes three times daily (08:00, 14:00, and 20:00), with feeding adjusted to ensure complete consumption within 10 min.

Experimental procedures followed the ARRIVE guidelines [77] for animal research and were approved by the Animal Ethics Committee of the Faculty of Biology, Alexandru Ioan Cuza University of Iași, Romania (Project approval no. 370/4 February 2022). All procedures complied with Directive 2010/63/EU of the European Parliament and Council on the protection of animals used for scientific purposes. No animals exhibited signs of toxicity or mortality during the study.

### 3.5. Group Allocation and Treatment

**Figure 8:**
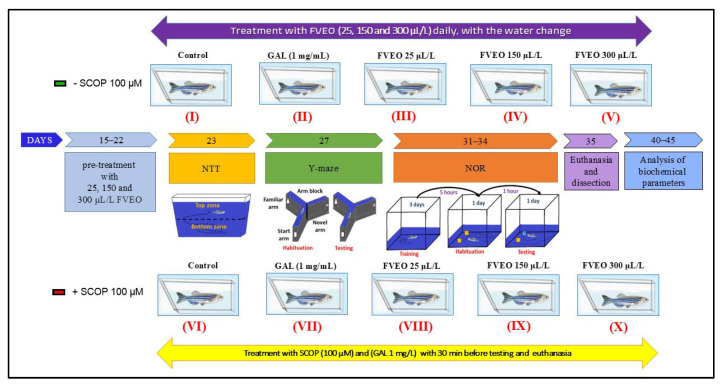
Schematic representation of experimental design. FVEO: *Foeniculum vulgare* Mill. essential oil; SCOP: scopolamine; GAL: galantamine; NTT: novel tank diving test; NOR: novel object recognition test.

Zebrafish were randomly assigned to 10 experimental groups (n = 10/group), as illustrated in Figure 8:

- **Group I (Control):** Untreated zebrafish maintained in standard conditions.
- **Group II (GAL):** Treated with galantamine (GAL, 1 mg/L), used as a positive control in behavioral and biochemical assessments.
- **Groups III–V (FVEO):** Treated with FVEO at 25, 150, and 300 µL/L, respectively.
- **Group VI (SCOP):** Treated with scopolamine (SCOP, 100 µM) to induce a dementia-like phenotype.
- **Group VII (SCOP + GAL):** Treated with SCOP (100 µM), followed by GAL (1 mg/L).
- **Groups VIII–X (SCOP + FVEO):** Treated with SCOP (100 µM), followed by FVEO at 25, 150, or 300 µL/L, respectively.

GAL (1 mg/L) was administered by immersion in a 500 mL cylindrical beaker for 3 min before behavioral testing. In Group VII, GAL was administered for 3 min following SCOP treatment. FVEO was chronically administered to Groups III–V and VIII–X at concentrations of 25, 150, or 300 µL/L dissolved in 1% Tween 80, with fresh solution provided during daily water changes. Dosages were selected based on previous findings [78]. The dementia-like state was induced using SCOP (100 µM, immersion for 30 min) before behavioral assessments, following protocols validated in previous studies [48,78]. This rigorously controlled protocol was designed to comprehensively evaluate the neuropharmacological and behavioral effects of FVEO in a validated zebrafish model of cognitive impairment.

### 3.6. Behavioral Analysis

To assess the effects of FVEO on zebrafish behavior, an advanced video tracking method was employed. Behavioral sessions were recorded using a Logitech HD Webcam C922 Pro Stream (Logitech, Lausanne, Switzerland). The recorded videos were subsequently analyzed using ANY-maze^®^ software version 7.48 (Stoelting Co., Wood Dale, IL, USA), which enabled automated and accurate quantification of behavioral parameters.

#### 3.6.1. Novel Tank Diving Test (NTT)

The NTT, a validated method for assessing anxiety-like behavior in zebrafish, was conducted as described by Cachat et al. [79]. The test apparatus consisted of a transparent trapezoidal tank (1.5 L; dimensions: 15.2 × 27.9 × 7.1 cm) divided equally into two horizontal zones: top and bottom. Following FVEO treatment, each fish was individually transferred to the testing tank, and behavior was recorded over a 6 min session.

Anxiety-like behavior was evaluated based on the following parameters: number of entries into the top zone, time spent in the top zone (s), and latency to first entry into the top zone (s). In addition, locomotor activity was assessed using distance traveled in the top zone (m), freezing duration (s), and average swimming velocity (m/s). These metrics provided comprehensive insights into both anxiety-related responses and general locomotor behavior, thereby facilitating a nuanced evaluation of FVEO’s neuropharmacological effects.

#### 3.6.2. Y-Maze Test

The Y-maze test was employed to assess the effects of FVEO on spatial memory and response to novelty in zebrafish, following the protocol described by Cognato et al. [80].

The apparatus consisted of a Y-shaped glass tank (3 L; arm dimensions: 25 × 8 × 15 cm), with distinct geometric shapes (squares, circles, triangles) affixed to the outer walls of each arm for spatial orientation. The three arms were designated as follows: (i) the start arm, from which the fish began each trial (always open); (ii) the novel arm, which was closed during the first trial and opened during the second; and (iii) the other arm, which remained open throughout both trials. The central intersection of the maze (neutral zone) was excluded from behavioral analysis.

The test consisted of two trials separated by a 1 h inter-trial interval. During the first trial (training phase, 5 min), zebrafish were allowed to explore only the start and other arms, with the novel arm blocked. In the second trial (test phase, 5 min), all three arms were accessible to evaluate exploratory behavior and memory performance. The following behavioral parameters were measured: total distance traveled (m), spontaneous alternation (%), turn angle (°), and time spent in the novel arm (s). These variables were used to assess spatial memory, locomotion, and response to novelty following FVEO treatment.

#### 3.6.3. Novel Object Recognition (NOR) Test

The NOR test was used to evaluate the effects of FVEO on recognition memory in zebrafish, following the protocol described by Stefanello et al. [81]. The experimental apparatus consisted of a 20 L glass tank (30 × 30 × 30 cm) filled with 6 cm of water.

Before testing, zebrafish underwent a habituation phase in which each animal was exposed to the empty tank for 5 min, twice daily (with a 5 h interval between sessions), over three consecutive days. On the fourth day, the training phase was conducted, during which each fish was individually exposed to two identical red cubes (familiar objects) placed in the tank for 10 min. Following a 1 h retention interval, the test phase was performed: one red cube (familiar object, F) was replaced with a green cube (novel object, N), and the fish were allowed to explore both objects freely for 10 min.

The behavioral parameters evaluated included the time spent exploring each object (s) and the percentage of preference for the novel object. The preference percentage was calculated using the formula: [Time exploring novel object/(Time exploring familiar + novel object)] × 100. This test allowed for the assessment of recognition memory and novelty discrimination following FVEO treatment.

### 3.7. Biochemical Parameters Assay

At the end of the behavioral testing, zebrafish were individually transferred to glass containers containing ice-cold water (2–4 °C) for 10 min to induce anesthesia. Euthanasia was then performed via rapid decapitation, following protocols previously described by our group [78]. Whole brains were immediately extracted, individually weighed (approximately 3–6 mg), and transferred to 0.5 mL microcentrifuge tubes. Samples were stored at −20 °C until further processing.

On the following day, brain samples from each experimental group were pooled and homogenized in cold phosphate buffer (0.1 M potassium phosphate, pH 7.4, containing 1.15% KCl) at a 1:10 (*w*/*v*) ratio. Homogenization was performed using a ball mill homogenizer (Mikro-Dismembrator U; Sartorius, NY, USA). The resulting homogenates were centrifuged at 14,000 rpm for 15 min at 4 °C. The supernatants were collected and used for subsequent biochemical assays.

To comprehensively evaluate the neuroprotective and antioxidant effects of FVEO on zebrafish, a suite of biochemical markers was assessed in brain tissue homogenates collected post-behavioral testing. These analyses targeted cholinergic function, oxidative damage, and redox homeostasis.

Total protein content was determined using the bicinchoninic acid (BCA) assay kit (Cat. No. B9643, Sigma-Aldrich, Darmstadt, Germany), and was used to normalize all enzymatic activities and biomarker concentrations.

AChE activity, an index of cholinergic function, was measured spectrophotometrically using Ellman’s method [82]. The reaction mixture (final volume: 1.5 mL) contained acetylthiocholine iodide (Cat. EC 3.1.1.7) and 5,5′-dithiobis(2-nitrobenzoic acid) (DTNB), both from Sigma-Aldrich. Samples were incubated at 37 °C for 15 min, and absorbance was recorded at 412 nm. AChE activity was expressed as nmol substrate hydrolyzed/min/mg protein.

SOD (EC 1.15.1.1) activity was determined by measuring the inhibition of nitro blue tetrazolium (NBT) photoreduction in the presence of riboflavin (Sigma-Aldrich, Germany) under fluorescent light [83]. The reaction mixture included 0.067 M potassium phosphate buffer (pH 7.8), 0.1 M disodium EDTA (Carl Roth, Germany), 1.5 mM NBT (AppliChem, Darmstadt, Germany), and 0.12 mM riboflavin, to which 50 µL of enzymatic extract was added for the test samples, while control samples contained no enzyme extract. The reaction mixtures were exposed to fluorescent light for 30 min at 25 °C, followed by spectrophotometric reading at 560 nm using a Beckman Coulter DU 730 Life Science UV-VIS spectrophotometer (Brea, CA, USA). SOD activity was expressed as units per milligram of protein (U/mg protein), where one unit represents the amount of enzyme required to produce 50% inhibition of NBT reduction.

CAT (EC 1.11.1.6) activity was assessed using a modified Sinha method [84] based on a chromogenic reaction with potassium dichromate and glacial acetic acid. A 0.1 M phosphate buffer (pH 7.4, with 1.15% KCl) served as the reaction medium. Hydrogen peroxide substrate solutions (0.16 M and 0.08 M) were freshly prepared in this buffer. Incubations were performed in a Thermomixer F1.5 (Eppendorf, Hamburg, Germany), and absorbance of the chromic acetate product was measured at 570 nm using a Beckman Coulter DU 730 UV-VIS spectrophotometer.

GPX (EC 1.11.1.9) activity was determined colorimetrically [85] by measuring the oxidation of reduced glutathione (GSH, Sigma-Aldrich, Germany) in the presence of hydrogen peroxide. The reaction mixture contained 0.25 M sodium phosphate buffer (pH 7.4), 25 mM EDTA, 0.4 M sodium azide, and 78 µL of enzyme extract, incubated for 10 min at 37 °C. The reaction was initiated by adding 50 mM GSH and 50 mM H_2_O_2_, followed by another 10 min incubation. The reaction was stopped with 7% metaphosphoric acid, centrifuged, and the supernatant was reacted with 0.3 M Na_2_HPO_4_ and DTNB. Absorbance was measured at 412 nm, and enzyme activity was expressed as µg GSH oxidized per mg protein.

GSH levels [86] were assessed using the DTNB-based method. Homogenates were deproteinized, neutralized, and incubated with DTNB. The yellow-colored complex was measured at 412 nm (final volume: 1.0 mL, 25 °C, 5 min). Results were reported as µmol GSH/mg protein.

Lipid peroxidation was estimated via MDA quantification using the thiobarbituric acid reactive substances (TBARS) assay [87]. Brain homogenates were incubated with thiobarbituric acid (TBA, Sigma-Aldrich) and perchloric acid (Merck, Darmstadt, Germany) in 1.5 mL reaction volume at 95 °C for 60 min. The MDA–TBA complex was measured at 532 nm and expressed as nmol MDA/mg protein.

Protein carbonyls were measured by precipitating 1 mg protein with 20% TCA, then reacting the pellet with 0.2% DNPH (Sigma-Aldrich) in 2N HCl for 1 h at room temperature [88]. After a second TCA precipitation and washing with ethanol/ethyl acetate (1:1), the pellet was dissolved in 6 M guanidine hydrochloride in phosphate buffer. Absorbance was read at 370 nm the next day, and results expressed as nmol DNPH/mg protein.

All reagents were of analytical grade and used according to manufacturer protocols.

### 3.8. Statistical Analysis

All data are presented as mean ± standard error of the mean (SEM) to reflect central tendency and variability. Statistical comparisons between experimental groups were conducted using one-way analysis of variance (ANOVA), followed by Tukey’s post hoc test for multiple comparisons, with treatment as the independent variable. Statistical significance was set at *p* < 0.05.

Analyses were performed using GraphPad Prism version 9.4 (GraphPad Software, Inc., San Diego, CA, USA). In addition, Pearson’s correlation coefficient (r) was used to explore potential relationships between behavioral parameters, enzymatic activity levels, and lipid peroxidation (MDA), offering insight into possible interdependencies within the biological response to FVEO treatment.

## 4. Conclusions

The results of this study demonstrate that FVEO, at concentrations of 25, 150, and 300 µL/L, exerts significant neuroprotective effects in a zebrafish model of cognitive impairment and anxiety. Notably, the lowest concentration (25 µL/L) showed the most pronounced behavioral improvements in cognitive and anxiety-related parameters, as assessed by the NTT, Y-maze, and NOR test.

FVEO effectively counteracted SCOP-induced oxidative stress in the brain, as evidenced by increased activity of antioxidant enzymes (SOD, CAT, and GPX), restoration of GSH levels, and reduction in protein carbonylation and lipid peroxidation (MDA). Furthermore, FVEO significantly inhibited AChE activity, supporting its role in enhancing cholinergic function and cognitive performance.

In silico ADMET analysis revealed favorable pharmacokinetic profiles for the five major FVEO compounds, including high absorption, efficient BBB and CNS permeability, and broad systemic distribution. Importantly, none of the compounds exhibited predicted hepatotoxicity, although a potential for epithelial tissue irritation was identified.

Together, these findings suggest that FVEO improves memory and anxiety-like behavior through dual modulation of the cholinergic system and oxidative balance in the brain. The results highlight the potential of FVEO as a natural therapeutic candidate for managing cognitive disorders and anxiety, offering a foundation for future studies on the role of essential oils in neuropharmacology.

## Figures and Tables

**Table 1:** Physicochemical properties of chemical compounds.

Descriptor	SCOP	GAL	*Trans*-Anethole	Camphor	*Trans*-Sabinene Hydrate	(+)-Carvone	*β*-Pinene
Molecular weight	303.358	287.359	148.20	152.23	154.253	150.22	136.23
No. heavy atoms	22	21	11	11	11	11	10
No. aroma heavy atoms	6	6	6	0	0	0	0
LogP	0.9181	1.8503	2.7283	2.4017	2.1935	2.4879	2.9987
Fraction csp^3^	0.59	0.53	0.20	0.90	1.00	0.50	0.80
Rotatable bonds	4	1	2	0	1	1	0
Acceptors	5	4	1	1	1	1	0
Donors	1	1	0	0	1	0	0
Molar refractivity	83.48	84.05	47.83	45.64	46.90	47.32	45.22
Surface area	129.371	124.520	67.315	68.174	68.806	67.800	63.322
TPSA	62.30 Å	41.93 Å	9.23 Å	17.07 Å	20.23 Å	17.07 Å	0.00 Å

**Table 2:** Prediction of intestinal absorption, P-glycoprotein substrate, P-glycoprotein I/II inhibitor, VDss, blood–brain barrier (BBB) permeability, CNS permeability, CYP3A4 substrate, CYP1A2 inhibitor, total clearance, renal OCT2 substrate, AMES toxicity, max. tolerated dose (human), oral rat acute toxicity (LD_50_), oral rat chronic toxicity (LOAEL), hepatotoxicity and skin sensitization.

Property	Compound Model Name	SCOP	GAL	*Trans*-Anethole	Camphor	*Trans*-Sabinene Hydrate	(+)-Carvone	*β*-Pinene	Unit
	Intestinal absorption (human) (low < 30%, high > 30%)	72.626	94.994	95.592	95.965	94.786	97.702	95.525	Numeric (% Absorbed)
	P-glycoprotein substrate	Yes	No	No	No	No	No	No	Categorical (Yes/No)
	P-glycoprotein I/II inhibitor	No	No	No	No	No	No	No	Categorical (Yes/No)
Distribution	VDss (human)	0.583	0.89	0.343	0.331	0.351	0.179	0.685	Numeric (log L/kg)
	Fraction unbound (human)	0.414	0.36	0.266	0.459	0.469	0.53	0.35	Numeric (Fu)
	BBB permeability (log BB > 0.3 cross BB, log BB < 0.1 do not coross BB)	−0.043	−0.081	0.529	0.612	0.663	0.588	0.818	Numeric (log BB)
	CNS permeability (log PS > −2, penetrate CNS, log PS < −3 do not penetrate)	−3.031	−2.511	−1.659	−2.158	−2.24	−2.478	−1.857	Numeric (log PS)
Metabolism	CYP3A4 substrate	Yes	Yes	No	No	No	No	No	Categorical (Yes/No)
	CYP1A2 inhibitor	No	No	Yes	No	No	No	No	Categorical (Yes/No)
Excretion	Total Clearance	1.096	0.991	0.268	0.109	1.011	0.225	0.03	Numeric (log ml/min/kg)
Toxicity	Max. tolerated dose (human) (low < 0.447, high > 0.477)	−0.319	−0.423	0.824	0.473	0.637	0.775	0.371	Numeric (log mg/kg/day)
	Oral Rat Acute Toxicity (LD50)	2.234	2.728	1.798	1.653	1.703	1.86	1.673	Numeric (mol/kg)
	Oral Rat Chronic Toxicity (LOAEL)	0.736	0.966	2.217	1.981	1.926	1.972	2.28	Numeric (log mg/kg_bw/day)
	Hepatotoxicity	No	Yes	No	No	No	No	No	Categorical (Yes/No)
	Skin Sensitisation	No	No	Yes	Yes	Yes	Yes	No	Categorical (Yes/No)

**Table 3:** Estimated pharmacokinetic parameters of drug-likeness and medicinal chemistry of the compounds analyzed in this study.

Drug-Likeness	SCOP	GAL	*Trans*- Anethole	Camphor	*Trans*- Sabinene Hydrate	(+)-Carvone	*β*-Pinene
Lipinski	Yes; 0 violation	Yes; 0 violation	Yes; 0 violation	Yes; 0 violation	Yes; 0 violation	Yes; 0 violation	Yes; 1 violation: MLOGP > 4.15
Ghose	Yes	Yes	No; 1 violation: MW < 160	No; 1 violation: MW < 160	No; 1 violation: MW < 160	No; 1 violation: MW < 160	No; 1 violation: MW < 160
Veber	Yes	Yes	Yes	Yes	Yes	Yes	Yes
Egan	Yes	Yes	Yes	Yes	Yes	Yes	Yes
Muegge	Yes	Yes	No; 2 violations: MW < 200, Heteroatoms < 2	No; 2 violations: MW < 200, Heteroatoms < 2	No; 2 violations: MW < 200, Heteroatoms < 2	No; 2 violations: MW < 200, Heteroatoms < 2	No; 2 violations: MW < 200, Heteroatoms < 2
Bioavailability Score	0.55	0.55	0.55	0.55	0.55	0.55	0.55

**Table 4:** Biological activity prediction of scopolamine, galantamine, and the major constituents of *Foeniculum vulgare* essential oil using PASS Online.

Drug-Likeness	SCOP		GAL		*Trans*-Anethole		Camphor		*Trans*-Sabinene Hydrate		(+)-Carvone		*β*-Pinene	
	*Pa*	*Pi*	*Pa*	*Pi*	*Pa*	*Pi*	*Pa*	*Pi*	*Pa*	*Pi*	*Pa*	*Pi*	*Pa*	*Pi*
ACh nicotinic antagonist	0.249	0.006	0.080	0.083	0.089	0.059	0.127	0.033	0.169	0.041	0.128	0.033	0.148	0.025
ACh neuromuscular blocking agent	0.351	0.171	0.376	0.154	0.594	0.025	0.674	0.006	0.483	0.085	0.729	0.004	0.571	0.035
Cholinergic antagonist	0.713	0.004	0.360	0.097	0.136	0.039	0.122	0.048	0.153	0.095	0.090	0.075	0.131	0.045
Neurotransmitter antagonist	0.396	0.095	0.736	0.003	0.549	0.024	0.498	0.041	0.410	0.086	0.336	0.146	0.464	0.056
Neurotrophic factor enhancer	0.113	0.252	0.176	0.102	0.176	0.102	0.498	0.041	0.252	0.029	0.155	0.140	0.202	0.066
Dementia treatment	0.285	0.164	0.458	0.026	0.499	0.015	0.574	0.005	0.540	0.008	0.332	0.105	0.537	0.009
Antineurotic	0.334	0.214	0.241	0.215	0.485	0.117	0.495	0.113	0.252	0.104	0.289	0.106	0.664	0.051
Antiinflammatory	0.338	0.130	0.140	0.115	0.526	0.049	0.252	0.116	0.839	0.005	0.473	0.065	0.611	0.029
Delirium	0.900	0.005	0.612	0.044	0.327	0.169	0.393	0.134	0.622	0.042	0.280	0.202	0.376	0.143
Behavioral disturbance	0.861	0.016	0.605	0.055	0.452	0.101	0.530	0.074	0.566	0.065	0.825	0.021	0.477	0.090
Depression	0.328	0.198	0.644	0.028	0.487	0.072	0.384	0.137	0.450	0.091	0.353	0.167	0.462	0.085
Lipid peroxidase inhibitor	0.521	0.015	0.369	0.025	0.439	0.026	0.242	0.098	0.239	0.100	0.465	0.067	0.070	0.059
NADPH peroxidase inhibitor	0.431	0.090	0.369	0.025	0.488	0.072	0.457	0.082	0.381	0.111	0.299	0.162	0.369	0.117

*Pa* (Active probability), *Pi* (Inactive probability).

## Data Availability

Data is contained within the article.

## References

[1] Scheltens P., Blennow K., Breteler M.M.B., de Strooper B., Frisoni G.B., Salloway S., Van der Flier W.M. (2016). Alzheimer’s Disease. Lancet.

[2] Graff-Radford J., Yong K.X.X., Apostolova L.G., Bouwman F.H., Carrillo M., Dickerson B.C., Rabinovici G.D., Schott J.M., Jones D.T., Murray M.E. (2021). New Insights into Atypical Alzheimer’s Disease in the Era of Biomarkers. Lancet Neurol..

[3] Hampel H., Mesulam M.M., Cuello A.C., Farlow M.R., Giacobini E., Grossberg G.T., Khachaturian A.S., Vergallo A., Cavedo E., Snyder P.J. (2018). The Cholinergic System in the Pathophysiology and Treatment of Alzheimer’s Disease. Brain.

[4] Chen Z.R., Huang J.B., Yang S.L., Hong F.F. (2022). Role of Cholinergic Signaling in Alzheimer’s Disease. Molecules.

[5] Bekdash R.A. (2021). The Cholinergic System, the Adrenergic System and the Neuropathology of Alzheimer’s Disease. Int. J. Mol. Sci..

[6] Fahimi A., Noroozi M., Salehi A. (2021). Enlargement of Early Endosomes and Traffic Jam in Basal Forebrain Cholinergic Neurons in Alzheimer’s Disease. Handb. Clin. Neurol..

[7] Martinez J.L., Zammit M.D., West N.R., Christian B.T., Bhattacharyya A. (2021). Basal Forebrain Cholinergic Neurons: Linking Down Syndrome and Alzheimer’s Disease. Front. Aging Neurosci..

[8] Lan T. (2023). Current Drug Treatments in Alzheimer’s Disease. Highlights Sci. Eng. Technol..

[9] Peitzika S.C., Pontiki E. (2023). A Review on Recent Approaches on Molecular Docking Studies of Novel Compounds Targeting Acetylcholinesterase in Alzheimer Disease. Molecules.

[10] Kandelshein H., Bloemer J. (2022). Side Effects of Drugs Used in the Treatment of Alzheimer’s Disease. Side Eff. Drugs Annu..

[11] Ilie A.C., Stefaniu R., Handaric M., Dascalescu S., Ivascu I., Alexa I.D. (2018). Adverse Effects Of Cholinesterase Inhibitors In The Elderly Patient With Myasthenia Gravis. Case Reports. Med.-Surg. J..

[12] Blokland A. (2022). Cholinergic Models of Memory Impairment in Animals and Man: Scopolamine vs. Biperiden. Behav. Pharmacol..

[13] Chen W.N., Yeong K.Y. (2020). Scopolamine, a Toxin-Induced Experimental Model, Used for Research in Alzheimer’s Disease. CNS Neurol. Disord. Drug Targets.

[14] Hsiao S.H., Hwang T.J., Lin F.J., Sheu J.J., Wu C.H. (2021). The Association Between the Use of Cholinesterase Inhibitors and Cardiovascular Events Among Older Patients With Alzheimer Disease. Mayo Clin. Proc..

[15] Hernández-Rodríguez M., Arciniega-Martínez I.M., García-Marín I.D., Correa-Basurto J., Rosales-Hernández M.C. (2020). Chronic Administration of Scopolamine Increased GSK3βP9, Beta Secretase, Amyloid Beta, and Oxidative Stress in the Hippocampus of Wistar Rats. Mol. Neurobiol..

[16] Kishore N., Verma A.K. (2022). Foeniculum Vulgare Mill: Flavoring, Pharmacological, Phytochemical, and Folklore Aspects. Medicinal Plants.

[17] Crescenzi M.A., D’Urso G., Piacente S., Montoro P. (2022). UPLC-ESI-QTRAP-MS/MS Analysis to Quantify Bioactive Compounds in Fennel (*Foeniculum Vulgare* Mill.) Waste with Potential Anti-Inflammatory Activity. Metabolites.

[18] Amiza A., Rauf A., ud Din A.M., Ahmad F., Sehar S., Khawaja A.A., Haroon S.M., Iqbal R. (2022). A Concise Review on Toxicity and Pharmacological Aspects of *Foeniculum Vulgare* with Emphasis on Anti-Cancer Potential. Asian J. Res. Pharm. Sci..

[19] Debnath S., Kumar H., Sharma A., Medicine I., Khoshnevisan K., Alipanah H., Baharifar H., Ranjbar N., Osanloo M. (2023). Foeniculum Vulgare from Spice to Pharma: Recent Advances in Its Medicinal Value, Bioactivities and Perspectives. Tradit. Integr. Med..

[20] Cherbal A., Bouabdallah M., Benhalla M., Hireche S., Desdous R. (2023). Phytochemical Screening, Phenolic Content, and Anti-Inflammatory Effect of Foeniculum Vulgare Seed Extract. Prev. Nutr. Food Sci..

[21] Patil J., Patil D., Sayyed H., Patil M., Mali R. (2022). Medicinal Traits of the Phenolic Compound from Foeniculum Vulgare for Oligomenorrhea. Chem. Proc..

[22] SwissADME. http://www.swissadme.ch/.

[23] Pires D.E.V., Blundell T.L., Ascher D.B. (2015). PkCSM: Predicting Small-Molecule Pharmacokinetic and Toxicity Properties Using Graph-Based Signatures. J. Med. Chem..

[24] van de Waterbeemd H., Gifford E. (2003). ADMET in Silico Modelling: Towards Prediction Paradise?. Nat. Rev. Drug Discov..

[25] Cioanca O., Hancianu M., Mircea C., Trifan A., Hritcu L. (2016). Essential Oils from Apiaceae as Valuable Resources in Neurological Disorders: Foeniculi Vulgare Aetheroleum. Ind. Crops Prod..

[26] Mareş C., Udrea A.M., Şuţan N.A., Avram S. (2023). Bioinformatics Tools for the Analysis of Active Compounds Identified in Ranunculaceae Species. Pharmaceuticals.

[27] Ibrahim M.T., Uzairu A. (2023). 2D-QSAR, Molecular Docking, Drug-Likeness, and ADMET/Pharmacokinetic Predictions of Some Non-Small Cell Lung Cancer Therapeutic Agents. J. Taibah Univ. Med. Sci..

[28] Dong J., Wang N.N., Yao Z.J., Zhang L., Cheng Y., Ouyang D., Lu A.P., Cao D.S. (2018). Admetlab: A Platform for Systematic ADMET Evaluation Based on a Comprehensively Collected ADMET Database. J. Cheminform..

[29] Lipinski C.A., Lombardo F., Dominy B.W., Feeney P.J. (1997). Experimental and Computational Approaches to Estimate Solubility and Permeability in Drug Discovery and Development Settings. Adv. Drug Deliv. Rev..

[30] Bickerton G.R., Paolini G.V., Besnard J., Muresan S., Hopkins A.L. (2012). Quantifying the Chemical Beauty of Drugs. Nat. Chem..

[31] Ghose A.K., Viswanadhan V.N., Wendoloski J.J. (1999). A Knowledge-Based Approach in Designing Combinatorial or Medicinal Chemistry Libraries for Drug Discovery. 1. A Qualitative and Quantitative Characterization of Known Drug Databases. J. Comb. Chem..

[32] Egan W.J., Merz K.M., Baldwin J.J. (2000). Prediction of Drug Absorption Using Multivariate Statistics. J. Med. Chem..

[33] Furey M.L., Khanna A., Hoffman E.M., Drevets W.C. (2010). Scopolamine Produces Larger Antidepressant and Antianxiety Effects in Women Than in Men. Neuropsychopharmacology.

[34] Hughes R.N., Otto M.T. (2013). Anxiolytic Effects of Environmental Enrichment Attenuate Sex-Related Anxiogenic Effects of Scopolamine in Rats. Prog. Neuropsychopharmacol. Biol. Psychiatry.

[35] Hamilton T.J., Morrill A., Lucas K., Gallup J., Harris M., Healey M., Pitman T., Schalomon M., Digweed S., Tresguerres M. (2017). Establishing Zebrafish as a Model to Study the Anxiolytic Effects of Scopolamine. Sci. Rep..

[36] Kim Y.H., Lee Y., Kim D., Jung M.W., Lee C.J. (2010). Scopolamine-Induced Learning Impairment Reversed by Physostigmine in Zebrafish. Neurosci. Res..

[37] Singsai K., Ladpala N., Dangja N., Boonchuen T., Jaikhamfu N., Fakthong P. (2021). Effect of Streblus Asper Leaf Extract on Scopolamine-Induced Memory Deficits in Zebrafish: The Model of Alzheimer’s Disease. Adv. Pharmacol. Pharm. Sci..

[38] Richetti S.K., Blank M., Capiotti K.M., Piato A.L., Bogo M.R., Vianna M.R., Bonan C.D. (2011). Quercetin and Rutin Prevent Scopolamine-Induced Memory Impairment in Zebrafish. Behav. Brain Res..

[39] Amoah V., Atawuchugi P., Jibira Y., Tandoh A., Ossei P.P.S., Sam G., Ainooson G. (2023). Lantana Camara Leaf Extract Ameliorates Memory Deficit and the Neuroinflammation Associated with Scopolamine-Induced Alzheimer’s-like Cognitive Impairment in Zebrafish and Mice. Pharm. Biol..

[40] Wang S., Su G., Zhang X., Song G., Zhang L., Zheng L., Zhao M. (2021). Characterization and Exploration of Potential Neuroprotective Peptides in Walnut (Juglans Regia) Protein Hydrolysate against Cholinergic System Damage and Oxidative Stress in Scopolamine-Induced Cognitive and Memory Impairment Mice and Zebrafish. J. Agric. Food Chem..

[41] Alvarado-García P.A.A., Soto-Vasquez M.R., Rosales-Cerquin L.E., Rodrigo-Villanueva E.M., Jara-Aguilar D.R., Tuesta-Collantes L. (2022). Anxiolytic and Antidepressant-like Effects of *Foeniculum Vulgare* Essential Oil. Pharmacogn. J..

[42] Raman S., Asle-Rousta M., Rahnema M. (2020). Protective Effect of Fennel, and Its Major Component Trans-Anethole against Social Isolation Induced Behavioral Deficits in Rats. Physiol. Int..

[43] Bahari N., Mahmoudi F., Haghighat K., Khazali H. (2023). The Effects of Trans-Anethole on the Hypothalamic CGRP and CRH Gene Expression in Rat Model of Stress. Arch. Adv. Biosci..

[44] Volgin A.D., Yakovlev O.A., Demin K.A., Alekseeva P.A., Kalueff A.V. (2019). Acute Behavioral Effects of Deliriant Hallucinogens Atropine and Scopolamine in Adult Zebrafish. Behav. Brain Res..

[45] Pande S., Patel C. (2023). Effect of Lactobacillus Rhamnosus and Diclofenac with Curcumin for Neuronal Restoration and Repair Against Scopolamine Induced Dementia in Zebrafish (*Danio Rerio*). Curr. Enzym. Inhib..

[46] Delaram E.E., Shahrbanoo O., Maryam K., Farhad V. (2019). Effect of Fennel Extract on the Improvement of Memory Disorders in Beta Amyloid Alzheimer Model of Male Wistar Rats. J. Ilam Univ. Med. Sci..

[47] Bhatti S., Ali Shah S.A., Ahmed T., Zahid S. (2018). Neuroprotective Effects of *Foeniculum Vulgare* Seeds Extract on Lead-Induced Neurotoxicity in Mice Brain. Drug Chem. Toxicol..

[48] Brinza I., Ayoub I.M., Eldahshan O.A., Hritcu L. (2021). Baicalein 5,6-Dimethyl Ether Prevents Memory Deficits in the Scopolamine Zebrafish Model by Regulating Cholinergic and Antioxidant Systems. Plants.

[49] Koppula S., Kumar H. (2013). *Foeniculum Vulgare* Mill (Umbelliferae) Attenuates Stress and Improves Memory in Wister Rats. Trop. J. Pharm. Res..

[50] Chang W., An J., Seol G.H., Han S.H., Yee J., Min S.S. (2022). Trans-Anethole Alleviates Trimethyltin Chloride-Induced Impairments in Long-Term Potentiation. Pharmaceutics.

[51] Richter N., Beckers N., Onur O.A., Dietlein M., Tittgemeyer M., Kracht L., Neumaier B., Fink G.R., Kukolja J. (2018). Effect of Cholinergic Treatment Depends on Cholinergic Integrity in Early Alzheimer’s Disease. Brain.

[52] Lane R.M., Potkin S.G., Enz A. (2006). Targeting Acetylcholinesterase and Butyrylcholinesterase in Dementia. Int. J. Neuropsychopharmacol..

[53] Abdel-Baki A.A.S., Aboelhadid S.M., Sokmen A., Al-Quraishy S., Hassan A.O., Kamel A.A. (2021). Larvicidal and Pupicidal Activities of *Foeniculum Vulgare* Essential Oil, Trans-Anethole and Fenchone against House Fly *Musca Domestica* and Their Inhibitory Effect on Acetylcholinestrase. Entomol. Res..

[54] Joshi H., Parle M. (2006). Cholinergic Basis of Memory-Strengthening Effect of *Foeniculum Vulgare* Linn. J. Med. Food.

[55] Shahriari M., Zibaee A., Sahebzadeh N., Shamakhi L. (2018). Effects of α-Pinene, Trans-Anethole, and Thymol as the Essential Oil Constituents on Antioxidant System and Acetylcholine Esterase of *Ephestia Kuehniella* Zeller (Lepidoptera: Pyralidae). Pestic. Biochem. Physiol..

[56] Smith M.A., Rottkamp C.A., Nunomura A., Raina A.K., Perry G. (2000). Oxidative Stress in Alzheimer’s Disease. Biochim. Biophys. Acta (BBA)-Mol. Basis Dis..

[57] Chen Z., Zhong C. (2014). Oxidative Stress in Alzheimer’s Disease. Neurosci. Bull..

[58] Huang W.J., Zhang X., Chen W.W. (2016). Role of Oxidative Stress in Alzheimer’s Disease (Review). Biomed. Rep..

[59] Muhammad T., Ali T., Ikram M., Khan A., Alam S.I., Kim M.O. (2019). Melatonin Rescue Oxidative Stress-Mediated Neuroinflammation/ Neurodegeneration and Memory Impairment in Scopolamine-Induced Amnesia Mice Model. J. Neuroimmune Pharmacol..

[60] Das M., Jaya Balan D., Kasi P.D. (2021). Mitigation of Oxidative Stress with Dihydroactinidiolide, a Natural Product against Scopolamine-Induced Amnesia in Swiss Albino Mice. Neurotoxicology.

[61] Alghamdi A., Al-Abbasi F.A., Alghamdi S., Alzarea S., Almalki W., Gupta G., Nadeem M., Sayyed N., Kazmi I., Alghamdi A.M. (2023). Europinidin Attenuates Scopolamine-Induced Deficit Memory in Rats by Improving Neurobehavioral Activity, Inhibiting AChE Levels and BDNF Expression. Authorea.

[62] Karthivashan G., Park S.Y., Kweon M.H., Kim J., Haque M.E., Cho D.Y., Kim I.S., Cho E.A., Ganesan P., Choi D.K. (2018). Ameliorative Potential of Desalted Salicornia Europaea L. Extract in Multifaceted Alzheimer’s-like Scopolamine-Induced Amnesic Mice Model. Sci. Rep..

[63] Yang Q., Lin J., Zhang H., Liu Y., Kan M., Xiu Z., Chen X., Lan X., Li X., Shi X. (2019). Ginsenoside Compound K Regulates Amyloid β via the Nrf2/Keap1 Signaling Pathway in Mice with Scopolamine Hydrobromide-Induced Memory Impairments. J. Mol. Neurosci..

[64] Zhang B., Zhao J., Wang Z., Xu L., Liu A., Du G. (2020). DL0410 Attenuates Oxidative Stress and Neuroinflammation via BDNF/TrkB/ERK/CREB and Nrf2/HO-1 Activation. Int. Immunopharmacol..

[65] Sun Z., Park S.Y., Hwang E., Park B., Seo S.A., Cho J.G., Zhang M., Yi T.H. (2016). Dietary *Foeniculum Vulgare* Mill Extract Attenuated UVB Irradiation-Induced Skin Photoaging by Activating of Nrf2 and Inhibiting MAPK Pathways. Phytomedicine.

[66] Yu C., Tong Y., Li Q., Wang T., Yang Z. (2022). Trans-Anethole Ameliorates Intestinal Injury Through Activation of Nrf2 Signaling Pathway in Subclinical Necrotic Enteritis-Induced Broilers. Front. Vet. Sci..

[67] Hashemi P., Ahmadi S. (2023). Alpha-Pinene Moderates Memory Impairment Induced by Kainic Acid via Improving the BDNF/TrkB/CREB Signaling Pathway in Rat Hippocampus. Front. Mol. Neurosci..

[68] Tripathi P., Tripathi R., Patel R.K., Pancholi S.S. (2013). Investigation of Antimutagenic Potential of *Foeniculum Vulgare* Essential Oil on Cyclophosphamide Induced Genotoxicity and Oxidative Stress in Mice. Drug Chem. Toxicol..

[69] Barakat H., Alkabeer I.A., Aljutaily T., Almujaydil M.S., Algheshairy R.M., Alhomaid R.M., Almutairi A.S., Mohamed A. (2022). Phenolics and Volatile Compounds of Fennel (*Foeniculum Vulgare*) Seeds and Their Sprouts Prevent Oxidative DNA Damage and Ameliorates CCl4-Induced Hepatotoxicity and Oxidative Stress in Rats. Antioxidants.

[70] Imran A., Xiao L., Ahmad W., Anwar H., Rasul A., Imran M., Aziz N., Razzaq A., Arshad M.U., Shabbir A. (2019). *Foeniculum Vulgare* (Fennel) Promotes Functional Recovery and Ameliorates Oxidative Stress Following a Lesion to the Sciatic Nerve in Mouse Model. J. Food Biochem..

[71] Ryu Y., Lee D., Jung S.H., Lee K.J., Jin H., Kim S.J., Lee H.M., Kim B., Won K.J. (2019). Sabinene Prevents Skeletal Muscle Atrophy by Inhibiting the MAPK–MuRF-1 Pathway in Rats. Int. J. Mol. Sci..

[72] Dhingra D. (2019). Sudha Antidepressant-Like Activity Of Trans-Anethole In Unstressed Mice And Stressed Mice. Asian J. Pharm. Clin. Res..

[73] Salama A., Mahmoud H.A.A.H., Kandeil M.A., Khalaf M.M. (2021). Neuroprotective Role of Camphor against Ciprofloxacin Induced Depression in Rats: Modulation of Nrf-2 and TLR4. Immunopharmacol. Immunotoxicol..

[74] Dai M., Wu L., Yu K., Xu R., Wei Y., Chinnathambi A., Alahmadi T.A., Zhou M. (2020). D-Carvone Inhibit Cerebral Ischemia/Reperfusion Induced Inflammatory Response TLR4/NLRP3 Signaling Pathway. Biomed. Pharmacother..

[75] Dahiya M., Kumar A., Yadav M. (2023). Ameliorative Effect of Β-pinene Targeting Mitochondrial Dysfunction and Oxidative Stress in Alzheimer’s Disease. Alzheimer’s Dement..

[76] Way2Drug-Main. https://www.way2drug.com/PASSOnline/index.php.

[77] Percie du Sert N., Hurst V., Ahluwalia A., Alam S., Avey M.T., Baker M., Browne W.J., Clark A., Cuthill I.C., Dirnagl U. (2020). The ARRIVE Guidelines 2.0: Updated Guidelines for Reporting Animal Research. PLoS Biol..

[78] Brinza I., Boiangiu R.S., Cioanca O., Hancianu M., Dumitru G., Hritcu L., Birsan G.-C., Todirascu-Ciornea E. (2023). Direct Evidence for Using Coriandrum Sativum Var. Microcarpum Essential Oil to Ameliorate Scopolamine-Induced Memory Impairment and Brain Oxidative Stress in the Zebrafish Model. Antioxidants.

[79] Stewart A., Cachat J., Wong K., Gaikwad S., Gilder T., DiLeo J., Chang K., Utterback E., Kalueff A.V. (2010). Homebase Behavior of Zebrafish in Novelty-Based Paradigms. Behav. Process..

[80] Cognato G.d.P., Bortolotto J.W., Blazina A.R., Christoff R.R., Lara D.R., Vianna M.R., Bonan C.D. (2012). Y-Maze Memory Task in Zebrafish (*Danio Rerio*): The Role of Glutamatergic and Cholinergic Systems on the Acquisition and Consolidation Periods. Neurobiol. Learn. Mem..

[81] Stefanello F.V., Fontana B.D., Ziani P.R., Müller T.E., Mezzomo N.J., Rosemberg D.B. (2019). Exploring Object Discrimination in Zebrafish: Behavioral Performance and Scopolamine-Induced Cognitive Deficits at Different Retention Intervals. Zebrafish.

[82] Ellman G.L., Courtney K.D., Andres V., Feather-Stone R.M. (1961). A New and Rapid Colorimetric Determination of Acetylcholinesterase Activity. Biochem. Pharmacol..

[83] Winterbourn C.C., Hawkins R.E., Brian M., Carrell R.W. (1975). The Estimation of Red Cell Superoxide Dismutase Activity. J. Lab. Clin. Med..

[84] Sinha A.K. (1972). Colorimetric Assay of Catalase. Anal. Biochem..

[85] Fukuzawa K., Tokumura A. (1976). Glutathione Peroxidase Activity in Tissues of Vitamin E-Deficient Mice. J. Nutr. Sci. Vitaminol..

[86] Salbitani G., Vona V., Bottone C., Petriccione M., Carfagna S. (2015). Sulfur Deprivation Results in Oxidative Perturbation in Chlorella Sorokiniana (211/8k). Plant Cell Physiol..

[87] Ohkawa H., Ohishi N., Yagi K. (1979). Assay for Lipid Peroxides in Animal Tissues by Thiobarbituric Acid Reaction. Anal. Biochem..

[88] Oliver C.N., Ahn B.W., Moerman E.J., Goldstein S., Stadtman E.R. (1987). Age-Related Changes in Oxidized Proteins. J. Biol. Chem..

